# Predictors for Emergency Admission Among Homeless Metastatic Cancer Patients and Association of Social Determinants of Health with Negative Health Outcomes

**DOI:** 10.3390/cancers17071121

**Published:** 2025-03-27

**Authors:** Poolakkad S. Satheeshkumar, Stephen T. Sonis, Joel B. Epstein, Roberto Pili

**Affiliations:** 1 Department of Medicine, Division of Hematology and Oncology, University at Buffalo, Buffalo, NY 14203, USA; rpili@buffalo.edu; 2 Divisions of Oral Medicine, Brigham and Women’s Hospital and the Dana-Faber Cancer Institute, Boston, MA 02115, USA; ssonis@biomodels.com; 3 City of Hope Comprehensive Cancer Center, Duarte, CA 91010, USA; jepstein@coh.org

**Keywords:** social determinants of health, emergency admissions, homelessness, problems related to living alone, prostate cancers, lung cancers, cancers of the lip, oral cavity, and pharynx, breast cancers, opioid abuse, burden of illness

## Abstract

Background/Objectives: Social determinants of health (SDOHs) are especially impactful with respect to emergency reliance among patients with cancer. Methods: To better predict the extent to which SDOHs affect emergency admissions in homeless patients with metastatic disease, we employed machine learning models, Lasso, ridge, random forest (RF), and elastic net (EN) regression. We also examined prostate cancer (PC), breast cancer (BC), lung (LC) cancer, and cancers of the lip, oral cavity, and pharynx (CLOP) for association between key SDOH variables—homelessness and living alone—and clinical outcomes. For this, we utilized generalized linear models to assess the association while controlling for patient and clinical characteristics. We used the United States National Inpatient Sample database for this study. Results: There were 2635 (weighted) metastatic cancer patients with homelessness. Transfer from another facility or not, elective admission or not, deficiency anemia, alcohol dependence, weekend admission or not, and blood loss anemia were the important predictors of emergency admission. C-statistics were associated with Lasso (train AUC-0.85; test AUC—0.86), ridge (85, 88), RF (0.96, 0.85), and EN (0.83, 0.80), respectively. In the adjusted analysis, PC homelessness was significantly associated with anxiety and depression (5.15, 95% CI: 3.17–8.35) and a longer LOS (1.96; 95% CI: 1.03–3.74). Findings were comparable in the BC, LC, and CLOP cohorts. Cancer patients with poor SDOHs presented with the worst clinical outcomes. Conclusions: Cancer patients with poor SDOH presented with worst clinical outcomes. The findings of this study highlight a vacuum in the cancer literature, and the recommendations stress the value of social support in achieving a better prognosis and Quality of life.

## Simple Summary

Cancer patients with adverse social determinants of health (SDOHs), specifically homelessness and living alone, in the prostate, breast, and lung categories, exhibited the most unfavorable clinical outcomes. These outcomes included heightened anxiety, depression, and extended hospital stays. SDOHs have a significant impact on the likelihood of emergency admissions among cancer patients. Machine learning algorithms are the most appropriate method for predicting the specific services needed by these patients.

## 1. Introduction

According to the Office of the Disease Prevention and Health Prevention, one of Healthy People 2030’s five overarching goals is related to social determinants of health (SDOHs): “Create social, physical, and economic environments that promote attaining the full potential for health and well-being for all.” [1]. SDOHs influence health and welfare—leading to health disparity and inequities—in the context of the social, environmental, economic, and cultural determinants of health [2]. In order to improve health outcomes and reduce enduring disparities in healthcare access, it is essential to comprehend the SDOHs in all disease scenarios, including cancer patients. As innovative treatment modalities become available, access to and influence on outcome research is mandatory [3]. The composition and dynamics of social networks, access to basic necessities like food, water, and shelter, as well as the quality of education, places of work, the security of their neighborhoods, all contribute to SDOHs [4]. Cancer patients show poor health outcomes when affected with challenging SDOHs, and recent reports indicate that SDOHs are associated with emergency admissions, hospital complications, and increased risk of cancer mortality [3,4,5,6,7]. Consequently, it is important to measure and quantify SDOHs in cancer patients.

National data identified 582,462 individuals reported to be homeless in the US in 2022. While the overall number of homeless persons increased little from 2020 to 2022, there was a dramatic spike in the numbers of persons without shelter, those with impairments, and those living alone who were at risk of homelessness [6]. Adults without children continue to make up the bulk of the homeless population, and the number of people sleeping on the streets has increased by 3.1% in the past year. The number of persons who have been without a home for a long period of time because of a handicap, known as chronically homeless people, increased by 16% from 2020 to 2022. Among the homeless, there is a disproportionate representation of persons of color (particularly Blacks, African Americans, and Africans) and indigenous peoples (including Native Americans and Pacific Islanders) [7]. The effects on the cancer cohort are unclear, despite the widespread knowledge that living alone is linked to a host of health problems [8,9,10]. On top of that, people with poor SDOHs are more likely to end up in the emergency room. At this time, we do not know what factors are to with emergency admissions among individuals with poor SDOHs [11]. In analyzing SDOHs, we examined the characteristics of cancer patients, which may influence cancer incidence, access to care, and health-seeking behaviors arising from societal challenges in the United States. Our hypothesis focuses on the influence of social and environmental factors, along with their interaction with individual traits, in sustaining racial and ethnic gaps in healthcare access and outcomes.

Thus, we assessed risk factors associated with emergency admission among metastatic cancer patients reporting two significant aspects of SDOHs—homelessness and living alone when diagnosed with cancers. Secondly, we focused on cancer cohorts, specifically LC, PC, BC, and CLOP to assess the impact of homelessness and living alone on clinical outcomes. Our objective in selecting several cancer cohorts is to elucidate the validity of our findings, as these malignancies emerge from distinct risk factors; hence, analyzing many cancers will yield a comprehensive understanding of their influence on cancer patients in the United States.

We utilized the National Inpatient Sample database to examine predictors associated with emergency admissions or services among metastatic cancer patients reported with homelessness. Furthermore, to assess differences in the risk and consequences of SDOHs—homelessness and problems related to living alone (PrbLA)—we assessed outcomes such as depression, anxiety, and length of in-hospital stay. Homelessness, a multifaceted social problem, is intricately connected to cultural frameworks, poverty, insufficient affordable housing, and structural disparities, resulting in stigma, discrimination, and obstacles to obtaining fundamental necessities and social integration. Conversely, PrbLA can substantially influence social dynamics, potentially resulting in social isolation and loneliness, which may adversely affect physical and mental health. Nonetheless, it is crucial to recognize that residing alone does not inherently imply loneliness or social isolation, since individuals can still preserve robust social connections. We assume that both variables are significant factors related to cancer patients. For this examination, we evaluated patients hospitalized for the treatment for PC, LC, BC, and CLOP. We also investigated the determinants of the primary pathway to hospital care among individuals with adverse SDOHs, a topic rarely explored in existing research. Consequently, our analysis in this area highlights the potential utility of machine learning analytics for future applications.

## 2. Methods

### 2.1. Study Design and Data Source

The Agency for Healthcare Research and Quality’s Healthcare Cost and Utilization Project (HCUP) National Inpatient Sample (NIS) database for 2017 was utilized. The NIS dataset includes patient sociodemographic and comorbidity information, as well as in-hospital outcomes, facility characteristics, and hospitalization charges [12].

### 2.2. Study Population

**Figure 1:**
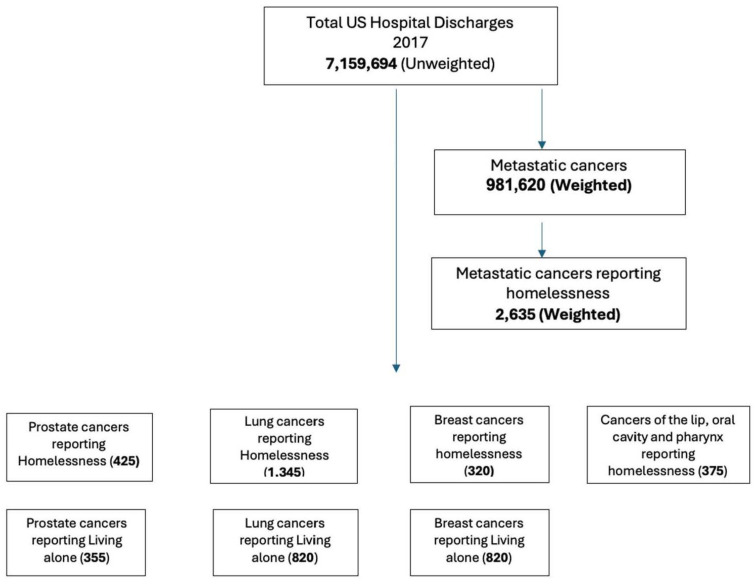
Flowchart (flowchart of the cancer cohort selection; sample size presented with weighted (original patient numbers) and unweighted numbers).

We used ICD-10-CM billing codes in our analysis for the following: types (PC, LC, and BC), key independent variables (homelessness [individuals without stable or dependable housing, attributable to poverty, insufficient affordable housing, mental illness, substance misuse, youth disconnection, or other circumstances (Figure 1). The ICD-10 code Z59.0 for homelessness is a medical classification designated by the WHO within the category of Factors Influencing Health Status and Contact with Health Services] and PrbLA [the ICD-10 code Z60.2 pertains to issues associated with living alone, classified by the WHO within the category of factors affecting health status and interactions with health services]), and outcomes of depression, anxiety, and illness burden (length of stay (LOS)). (In the supplementary file, ICD codes are included). Emergency services/admissions are provided in the dataset, and metastatic cancers are provided in the co-morbidity file obtained through the Elixhauser comorbidity index.

### 2.3. Study Measurements

For the prediction model, we stratified metastatic cancer patients reported with homelessness. For the study of the association of clinical outcome and SDOHs, the key independent variables included homelessness and PrbLA among patients with PC, LC, and BC, and the outcome variables comprised depression, anxiety, and length of in-hospital stay. The term “total charges” was used to refer to the total cost of all healthcare services related to the hospital admission, excluding professional medical services. In-hospital mortality is defined as death that occurs while a patient is receiving medical attention and is recorded as either alive or dead at the time of discharge. Characteristics at the patient and clinical levels were added as covariates. Age, sex, race (White, Black, Hispanic, Asian, and others), primary payer (Medicare, Medicaid, private insurance, and others), median household income based on zip code (first to fourth quartile), and patient location (urban/rural—using a six-category urban–rural classification scheme for the United States’ counties developed by the National Center for Health Statistics) were all included as patient characteristics in the study. Clinical characteristics included admission origin (transferred in, not transferred), admission type (elective vs. non-elective; elective indicates whether patients were voluntarily hospitalized), and the Elixhauser comorbidity index, which was used to classify comorbidities.

### 2.4. Statistical Analysis

#### 2.4.1. Prediction Models and Risk Factor Analysis

For subset selection in constructing the ML model, we used forward selection and backward elimination regression techniques, with the minimal Akaike information criterion (AIC) being used to determine the best subsets. Important variables were identified through forward selection and backward removal, and those results were used to construct ML models [13,14]. In addition, we used the Boruta package [15], and gradient boosting machine (GBM) to obtain and compare the feature selection models. The features which optimized model performance were utilized for the ML model building.

Subsequently, we used both linear and non-linear ML techniques to construct our models. The least absolute shrinkage and selection operator (Lasso), ridge, and elastic net regression were used as the linear model, and RF was used as the non-linear model. Both the training set and the test set were used to evaluate the lasso regression model’s efficacy via cross validation. The model was trained with 70% of the data and validated with the remaining 30%. We also used receiver operator curves (ROCs) and the Hosmer–Lemeshow test to compare the model’s performance on both the training set and the test set. The same processes were repeated for the ridge and EN regression. Similarly, we used random forest (RF) to estimate the ML model’s prediction and explainability (Supplementary Materials). Classification accuracy was also measured using the ROCs, Hosmer–Lemeshow test, and confusion matrix.

Additionally, we used RF for employing the explainable artificial intelligence (XAI); the process of parameter tuning was included with elaborate computation through a parallel core through the use of “detectCores”, utilized to automatically determine the number of cores for the “makePSOCKcluster” functions [16,17,18,19]. We additionally used Local Interpretable Model-Agnostic Explanations (LIME) techniques as a means of achieving local interpretation [20]. R 4.6.2 was used for all statistical testing (R Foundation for Statistical Computing, Vienna, Austria).

#### 2.4.2. Association with Outcomes

Each cohort’s demographic and clinical features were described using descriptive statistics. Due to the non-normal distribution of total charges and LOS, we log-transformed these data and provided the geometric mean. A value of 0.0001 was imputed for a LOS of 0 days to prevent a negative log.

Univariate analysis, by scrutinizing individual variables, facilitated the identification of potential predictors and understanding their attributes, thus informing the selection of variables for more complex multivariate analyses, ensuring a more focused and efficient approach while establishing a foundational basis for variable selection. This enabled the understanding of variable distributions through the analysis of their distribution, central tendency, and dispersion, serving as a basis for variable selection, recognizing outliers and anomalies, clarifying intricate relationships (such as race/ethnicity and SDOHs), intentional variable selection, and verifying assumptions. Additionally, we used survey-weighted generalized linear models to examine the relationship between each cohort’s outcomes (depression, anxiety, length of hospital stay, and total charge) and the exposure status (either homelessness or living alone). Both univariate and multivariate Svyglm were employed to investigate the relationship between important independent variables and the outcome in the two patient cohorts. Age, gender, payer type, patient location, race, and median household income, as well as other factors like the co-morbidity score and whether or not the patient was transferred out of the hospital were all taken into account when adjusting the multivariable Svyglm models. We utilized a family reference quasibinomial for fitting the Svyglm to the binary dichotomous outcome models. We used two-tailed probability distributions for all of our studies, and *p* < 0.05 was used to define statistical significance. The R 3.6.3 statistical computing environment (R Foundation for Statistical Computing, Vienna, Austria) was used to perform all statistical analyses.

## 3. Results

### 3.1. Prediction Model and Risk Factors

There were 2635 (weighted) metastatic cancer patients reporting homelessness, with a mean [SD] age of 56.8 [9.94]; females were reported at only 26.4%, 46% of homeless individuals stated they were living in low-income neighborhoods, and 85% were either Medicare or Medicaid beneficiaries (Figure 1). Federal programs such as Medicare allocate greater funds for the treatment of homeless patients, and the federal government acknowledges homelessness as a social predictor of health. This modification seeks to enhance patient outcomes including safety and health equity, whereas the additional state-led Medicaid programs are customized for each state, reflecting the unique demographics, healthcare systems, needs, and financial limitations of that states. And 77.4% of them had sought emergency services (Table 1). Features identified from feature selection backward elimination and forward selection in addition to the Boruta included transferred in or not from another facility, elective admission or not, deficiency anemia, alcohol dependence, weekend admission or not, and blood loss anemia, which were the important predictors of emergency admission for metastatic cancer patients reported with homelessness. For building the linear and non-linear models, we additionally used race, payer status, median household income based on zip code, weight loss, and fluid and electrolyte imbalance as predictors along with the feature selection variables. These additional variables provided a best fit and explanation for the ML and XAI approaches. Multiple iterations were made for a final selection of variables for building ML models.

**Figure 2:**
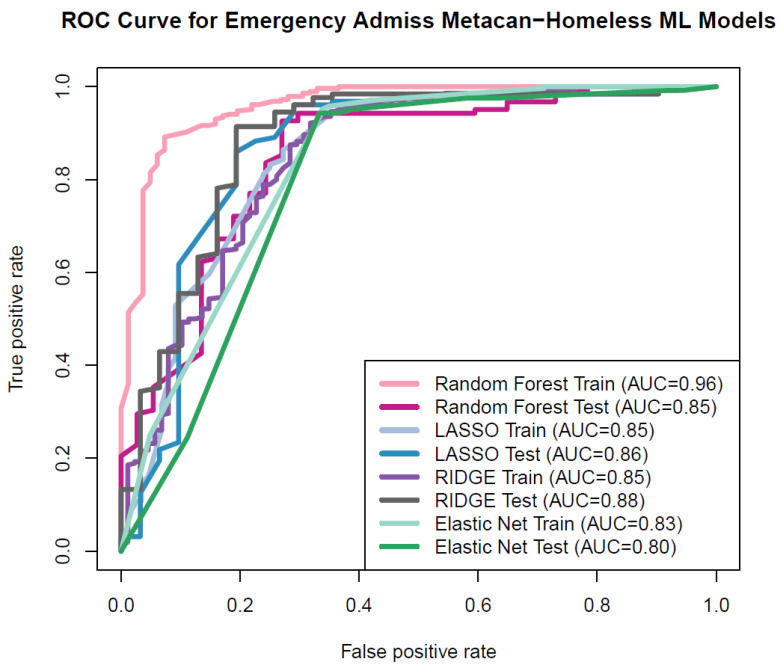
ROC curve for prediction model.

The non-linear RF model showed excellent discrimination performance in predicting the emergency room admissions. Linear models, specifically Lasso, ridge and elastic net regression, demonstrated very good performance in the ML models. The area under the receiver operating characteristic (ROC) curve (area under the curve, AUC) had values of 0.85 for testing and 0.86 for training for Lasso and 0.85 for testing and 0.88 for testing data for ridge, respectively. For the EN regression, the AUC for training was 0.83 and for testing, it was 0.80. Furthermore, for random forest (RF), it was 0.96 for training and 0.85 for testing data (Figure 2). Consequently, the area under the receiver operating characteristic (ROC) curve serves as the common performance metric for both the training and testing datasets in the context of linear and non-linear ML methods.

**Figure 3:**
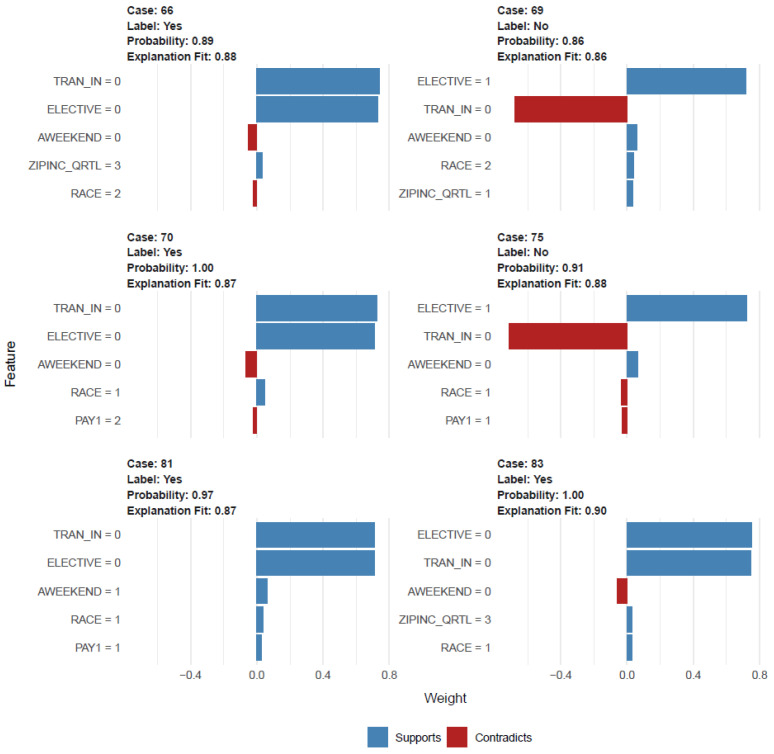
XAI (explainable artificial intelligence)-based plot.

RF XAI-based explainability was constructed with 368 samples (unweighted), 12 predictors, and two classes of “No” and “Yes”. The confusion matrix and statistics showed the following: accuracy: 0.86, 95% CI: (0.79, 0.91), *p*-value [Acc > NIR]: 0.002, kappa: 0.58. And the explanation plot shows the cases from 66 to 83, with predicted root variables transferred in and elective admission along with race, median household income, payer status, and weekend admission. The predicted probability ranged from 0.86 (case 69) to 1.00 (case 83) with an explanation fit of 0.86 (case 69) to 0.99 (case 83) (Figure 3).

### 3.2. PC Cohort and Homelessness

There were 209,410 PC patients and among them, there were 425 patients who reported homelessness. (Table 2). The mean age of the homeless PC patients was five years younger than the non-homeless PC patients (71.5 vs. 64.6). The highest number of patients with homelessness was seen in the age group of 55–64, and this was lower among the >65 age group. Homelessness was higher among Black people, and the primary payer for the homeless group was mostly Medicare and Medicaid. We noted that PC homeless individuals reported having higher emergency room admissions, opioid abuse, and opioid long-term use.

PC homeless patients were associated with higher combined anxiety and depression (unadjusted (coefficient, 4.06; 95% CI: 2.61–6.33), adjusted (5.15, 95% CI: 3.17–8.35)), as well as a longer LOS (unadjusted (2.49; 95% CI: 1.58–3.92), adjusted analysis (1.96; 95% CI: 1.03–3.74)) (Table 3).

### 3.3. PC Cohort and PrbLA

Among 209,410 PC patients, there were 355 patients who reported PrbLA. The mean age of the PrbLA PC patients was five years older than the non-homeless PC patients (71.5 vs. 76.1) (Table 2). The primary payer for the PrbLA group was Medicare. We noted that PC PrbLA patients reported having higher emergency room admissions and opioid long-term use. PC patients with PrbLA were associated with higher combined anxiety and depression (unadjusted (coefficient, 2.77; 95% CI: 1.69–4.53), adjusted (2.7, 95% CI: 1.64–4.46)) and a longer LOS (unadjusted (2.08; 95% CI: 1.63–2.68), adjusted analysis (1.44; 95% CI: 1.11–1.88)) (Supplementary Table S1).

### 3.4. BC Cohort and Homelessness

There were 117,080 BC patients and among them, there were 320 patients who reported homelessness. The mean age of the homeless BC patients was five years younger than the non-homeless BC patients (61.7 vs. 54.7). Most of the BC homeless patients belonged to the age groups of 45–54 and 55–64, and the lowest were among the >65 age group (Table 4). Most of the homeless BC patients had Medicare and Medicaid payer status. We noted that BC homeless patients reported having higher emergency room admissions and opioid abuse.

BC homeless patients were associated with higher anxiety (unadjusted (coefficient: 2.07; 95% CI: 1.19–3.63), adjusted (2.07, 95% CI: 1.06–4.03)) (Table 5).

### 3.5. BC Cohort and PrbLA

There were 117,080 BC patients and among them, there were 225 patients who reported PrbLA. The mean age of the homeless BC patients was nine years older than the non-PrbLA BC patients (61.7 vs. 54.7). Most of the BC PrbLA patients were in the 55–64 and >65 age groups (Table 4). The majority of the PrbLA BC patients had Medicare and private insurance, and most of them were transferred out to a different care facility for aftercare. We noted that BC PrbLA patients reported having higher anxiety and depression and opioid long-term use. BC patients with PrbLA were associated with higher depression (unadjusted (coefficient, 2.99; 95% CI: 1.64–5.45), adjusted (3.19, 95% CI: 1.63–6.25)); combined anxiety and depression (unadjusted (coefficient 1.94; 95% CI: 1.05–3.59), adjusted (2.19, 95% CI: 1.12–4.29)); and a longer LOS (unadjusted (2.32; 95% CI: 1.69–3.2), adjusted (2.14, 95% CI: 1.63–2.82)) (Supplementary Table S2).

### 3.6. LC Cohorts and Homelessness

There were 403,010 LC patients and among them, there were 1345 patients who reported homelessness. The mean age of the homeless LC patients was nine years younger than the non-homeless lung cancer patients (60.4 vs. 69.1). Females with LC reported less homelessness compared to males. The LC homeless patients represented a higher proportion among the age groups of 45–54 and 55–64, and this was lower among the >65 age group. (Table 6) Similarly to the PC and the BC homeless patients, homelessness was higher among Black individuals, and the primary payer for the homeless group was mostly Medicare and Medicaid. We noted that LC homeless patients reported having higher opioid abuse. LC homeless patients were associated with higher combined anxiety and depression (unadjusted (coefficient: 1.4; 95% CI: 1.08–1.83), adjusted (1.37, 95% CI: 1.02–1.85)) and higher LOS (unadjusted (2.08; 95% CI: 1.6–2.71), adjusted analysis (1.84; 95% CI: 1.4–2.42)) (Table 7).

### 3.7. LC Cohorts and PrbLA

Among 403,010 LC patients, there were 820 patients who reported PrbLA. The mean age of the PrbLA LC patients was three years older than the non-PrbLA LC patients (69.1 vs. 72.2). As compared to males, females were reported with more PrbLA. LC PrbLA patients represented a higher proportion among the >65 age group (Table 6). The primary payer for the PrbLA group was mostly Medicare and private insurance (78% and 12%). LC PrbLA patients were not associated with anxiety and depression, nor LOS. However, when stratified to females only, LC PrbLA patients had a longer LOS in the unadjusted (1.30; 95% CI: 1.04–1.64) and in the adjusted analysis (1.29; 95% CI: 1.02–1.63) (Supplementary Table S3).

### 3.8. CLOP Cohorts and Homelessness

There were 53,890 CLOP patients and among them, there were 375 patients who reported homelessness. The mean age of the homeless CLOP patients with homelessness was 10 years younger than the non-homeless lung cancer patients (64.06 vs. 54.85). Females with CLOP reported less homelessness compared to males. The CLOP homeless patients represented a higher proportion among the 45–54 and 55–64 age groups, and this was lower among the >65 age group (Table 8). Similarly to the PC, LC, and BC homeless patients, homelessness was higher among Black individuals, and the primary payer for the homeless group was mostly Medicare and Medicaid. We noted that CLOP homeless patients reported having higher emergency admissions. CLOP homeless patients were associated with higher combined anxiety and depression (unadjusted (coefficient—2.42; 95% CI: 1.52–3.85), adjusted, 2.80 (95% CI: 1.68–4.69)) and a higher LOS (unadjusted (coeff: 3.19; 95% CI: 2.8–4.45), adjusted analysis (coeff: 1.96; 95% CI:1.03–3.74)) (Supplementary Table S4). Moreover, the CLOP lacked critical PrbLA data for the association investigation.

### 3.9. Factors Associated with Risk of Homelessness and PrbLA and the Outcome of Anxiety and Depression and LOS Among PC, LC, BC, and CLOP Cancer Patients

In the PC cohort, when compared to Whites, homelessness was associated with higher emergency department visits but lower anxiety and depression among Blacks, Hispanics, Asians, and others. Furthermore, when compared to Whites, Blacks alone had a longer LOS. Homelessness was also associated with fewer emergency department visits among middle- and high-income neighborhoods compared to low-income neighborhoods (Table 3). Blacks, Hispanics, Asians, and others had less anxiety and depression compared to Whites when associated with PrbLA, and Blacks had a higher LOS compared to Whites (Supplementary Table S1).

In the LC cohort, when compared to Whites, homelessness was associated with less anxiety and depression among Blacks, Hispanics, and others. And in the LC cohort, when compared to Whites, homelessness was also associated with a longer LOS among Blacks, Asians, Native Americans, and others. Furthermore, when compared to low-income neighborhoods, homelessness was associated with a shorter LOS among middle- and high-income neighborhoods. (Table 7). The LC PrbLA group, when stratified for females, was associated with a longer LOS, and Blacks had a longer LOS compared to Whites (Supplementary Table S3). In the PC and LC cohorts, compared to Medicare, those with private insurance had less anxiety and depression, as well as a shorter LOS (Supplementary tables).

In the BC cohort, when compared to Whites, homelessness was associated with less anxiety among Blacks and Hispanics, as well as Asians, Native Americans, and others. Compared to Medicare, homelessness was associated with anxiety among those with private insurance. The BC PrbLA group was associated with anxiety and depression and a longer LOS (Supplementary Table S2). Compared to Whites, Blacks, Hispanics, Asians, and others had anxiety and depression. However, Blacks had a longer LOS compared to Whites. In the CLOP cohort, compared to males, females with homelessness were associated with more anxiety and depression (Supplementary Table S4).

## 4. Discussion

The primary predictors linked to emergency admissions in metastatic cancer patients experiencing homelessness include deficiency anemia, alcohol dependence, weekend admissions, blood loss anemia, and transfer status from other facilities. Along with these root nodes/variables of feature selection, we utilized race, payer status, median household income based on zip code, alcohol dependence, weight loss, and fluid and electrolyte imbalance. The prediction of emergency admission utilizing ML methods showed excellent prediction in the RF training model and RF testing model, as well as in the linear models of Lasso, ridge, and EN regression.

In this study, PC, BC, LC, and CLOP patients with poor SDOHs—homelessness and PrbLA—are associated with anxiety and depression and a longer in-hospital LOS. Additionally, Blacks and those living in low-income neighborhoods are disproportionally affected by SDOHs. Whites reported higher anxiety and depression levels in all cancer cohorts.

SDOHs are a composite set of conditions affecting the individual’s outcome performance in overall health and welfare [21]. There are five major determinants, namely economic stability, education access and quality, healthcare access and quality, the neighborhood and built environment, and social and community context [22]. However, the SDOH in cancer is not well explored; nevertheless, the Patient Protection and Affordable Care Act (PPACA) in 2010 subsequently led to initiatives to develop policies, roadmaps, and federal and non-federal programs [23,24,25,26,27,28]. Even after 14 years of the PPACA, there has been slow progress in cancer setting in assessing measures affecting cancer care from screening to cancer survivorship care. Considering homelessness, there has been a remarkable achievement in a 2017 report, illustrating that the number of chronically homeless people in America has decreased by 22,892 (or 21 percent) since 2010. Furthermore, a decrease of 25 percent, or 10,565 people, in the number of chronically homeless persons who are in shelters since 2007 has been observed, as well as a decrease of 33 percent, or 25,632 people, in the number of chronically homeless people who are living on the streets [29]. However, a recent report (2022) indicates that the 2017 homelessness progress has been interrupted across the country, primarily affected by COVID-19, and its economic impact and public policy has led to a significant increase in homelessness in most places [6]. Despite a small increase from 2020 to 2022 in the overall number of homeless persons, the numbers of unsheltered people, people with disabilities, and long-term homeless people all grew significantly. The majority of persons who are homeless are single people without secure homes, and the percentage of single homeless people increased by 3.1%. Furthermore, the number of persons who were chronically homeless, which includes those who were disabled and had been homeless for an extended time, increased by 16 percent between 2020 and 2022 [6]. Furthermore, these patients suffer from chronic illnesses and require emergency admission. They also experience additional problems related to hospitalization, which contribute to poorer outcomes in any given medical situation [2,3,4,5,6,7].

Given the various factors that impact a patient’s choice to be admitted, accurately predicting emergency admissions in any group is exceedingly challenging. However, precisely predicting emergency hospitalization among patients with poor SDOHs is particularly difficult due to the added complexities arising from factors such as decision-making, need, payer status, and healthcare accessibility. However, with the help of advanced algorithms specifically designed for technology, a variety of technology-focused considerations and procedures can be evaluated, examined, and successfully implemented in these settings [30,31,32,33]. Through the analysis of yearly national data that are gathered and maintained electronically, we were able to use algorithm-specific validation in our study to predict emergency admissions among homeless metastatic cancer patients using ML XAI. Thus, it is implied that there is a commonality in the pattern of care, living circumstances, access to healthcare, and behavior related to seeking health among homeless metastatic cancer patients. Hence, all of our models, including Lasso, ridge, EN, and RF, performed well in prediction using the variables selected by the machines. These variables include whether the patient was transferred from another hospital, whether the admission was elective or not, the presence of deficiency anemia, alcohol dependence, weekend admission status, and the presence of blood loss anemia. In addition to these primary nodes/variables for feature selection, we incorporated multiple iterations to include race, payer status, median household income determined by zip code, weight loss, and fluid and electrolyte imbalance, and thus yielded a better performance in prediction among poor SDOHs. Recent data indicate that there have been 4 million emergency services provided for oncology patients [34,35,36]. This increase in emergencies can be attributed to the increasing complexity and the widespread use of active therapy, which have resulted in compromised conditions among advanced cancer patients; however, the access to care, payor status, decision-making, need, and acute care while being chronic patients all affect this outcome [37]. Our data show that there is a much higher rate of emergency admissions (70~80%) among all cancer cohorts who report being homeless. This suggests that there is a greater unmet need among those experiencing poor SDOHs, and this trend aligns with findings from other research studies [38].

Apart from the impact of access to specialty services [39], need, complications, and family influences [40,41], there is also a relationship between SDOHs and whether or not a patient transfers in. Non-elective and weekend admission are equally indicative of unfavorable health in these populations, as is the combination of demographics, race, and income statuses from the findings.

The current research on providing private healthcare to the homeless, including individuals experiencing episodic poverty, chronic or long-term poverty, and residing in impoverished areas, is encouraging, largely due to the rising role of respite and recuperative care in various states nationwide [42,43,44,45,46]. In our analysis, across all cancer cohorts, homeless patients received support at 6 to 8% from private insurance, with the majority of assistance coming from Medicare and Medicaid programs (80–90%). The recent CHIP program by Medicaid is an attractive option for respite and recuperative care, jointly financed by state and federal governments [47].

Research has examined the impact of social networks on homelessness and its implications for racial and ethnic backgrounds that affect pro-social behaviors and susceptibility to substance misuse among younger populations [48,49,50]. During the examination of homelessness, all cohorts were younger than their non-homeless counterparts; conversely, cancer patients with PrbLA were older than those without PrbLA. Moreover, throughout all cancer cohorts, homeless cancer patients primarily resided in low-income districts, but they were also observed to inhabit middle- and high-income neighborhoods. A longitudinal evaluation of homeless individuals conducted by Alexander-Eitzman et al. reveals that their sleeping locations varied within urban centers, exhibiting a spatial distribution pattern. Over a span of two years, these individuals transitioned from areas of greater housing and economic distress, as well as material deprivation, to locations with comparatively less hardship [51].

Homelessness correlates with less cancer screening; a report from the PC cohort also indicates that screening and the presence of a primary care physician were predominantly influenced by homelessness in the adjusted analysis [52]. The research on cancer patients experiencing homelessness is scarce, highlighting an understudied topic that requires attention, covering aspects such as survivability, hospice access, and the availability of food, medications, and housing [53]. The homelessness of cancer patients is further compounded by literacy rates [54], continuity of care [55,56], access to resources [57,58,59], and outcomes related to tobacco, alcohol, and drug toxicity and mortality [60]. This study noted that all cancer cohorts with unfavorable SDOHs were linked to elevated chances of opioid misuse, emergency department visits, heightened anxiety and depression, and longer lengths of stay in the adjusted analysis. Substance misuse and overdose are prevalent issues among the homeless population, with a consistent bidirectional association [61,62,63]. Substance usage rates among the homeless are projected to exceed those of the general population, although these percentages may fluctuate significantly according on the specific group examined and the categories utilized [64,65]. The intricacies of substance misuse, interactions with cancer therapy, anxiety, depression, and emergency room dynamics remain poorly defined. The majority of fatalities among the homeless can be attributed to substance misuse, with drug overdoses as the predominant cause of death [66,67]. To successfully tackle these interconnected challenges, it is essential to comprehend the relationships among homelessness, substance misuse, anxiety, and depression.

Not surprisingly, we observed that, in general, SDOHs were associated with worse outcomes among Black cancer patients. Interestingly, the mean age of the homeless cancer patients was ~five years younger than the non-homeless cancer patients, suggesting a more aggressive natural history in this patient population. We found that homelessness was associated with less anxiety among Blacks and Hispanics compared to Whites. This observation should provide a rationale for further research. The severity and effects of poor SDOHs tend to be worse for cancer patients, and it is highly desirable to develop mixed methods to assess the unmet need among them. This research included only in-patient cohorts, examining outpatient and ambulatory care are other significant assignments and are critically needed. Furthermore, the likelihood of having cancer in the realm of poor SDOHs and the likelihood of having other conditions—co-morbidities, poor nutritional status, and substance abuse—may lead to a multitude of complex disease patterns affecting survival. To enhance patient outcomes in cancer care, it is crucial to document or assess the social determinants of health (SDOHs), which encompass factors from severe poverty to social isolation. Additionally, implementing strategies to deliver superior care to individuals facing adverse SDOHs is essential for improving outcomes in cancer treatment, including survivorship care.

Considerations for the homelessness group and variety of payer groups, as well as cost implications, include the following:

1. While certain homeless individuals may seek treatment in hospitals either ambulatory or in-hospital admission care, others become ensnared in a detrimental cycle of homelessness and hospitalization [68,69]; nevertheless, this may not apply to cancer patients. However, the period preceding homelessness is associated with an increased likelihood of hospitalization [70]. Research shows that medical emergencies and SDOHs are associated with emergency admissions [70,71]. Furthermore, among cancer patients, this might be exacerbated by SDOHs and predictive factors such as transferred in or not from another facility, elective admission or not, deficiency anemia, alcohol dependence, weekend admission or not, and blood loss anemia. Additional research is needed to identify the outcome of infectious complications and healthcare-associated complications in this cohort that additionally impact the burden of illness among cancer patients.
2. There is insufficient study on integrating data on homelessness with hospitalized or ambulatory care systems for cancer patients. Research on the financial demands and healthcare consumption patterns of cancer patients is limited, and the existing studies often concentrate on certain demographics or service categories. The current study indicates that patients utilized Medicaid or Medicare services more frequently before and after their enrollment in shelters, motels, relative housing, or their own residences, following recent housing insecurity that likely contributed to homelessness. Upon analyzing Medicaid, Medicare, and income-based housing status, it was found that residency in neighborhoods containing shelters, motels, relative housing, or personal housing—regardless of employment status—was frequently mandated.
3. A deeper comprehension of the correlation between homelessness in cancer patients and healthcare systems should guide future initiatives. The use of hospitals during periods of homelessness may reveal the potential role of health systems in preventing or alleviating homelessness, the effects of housing instability on health and healthcare utilization, and the distinct implications of homelessness on healthcare consumption compared to standard hospitalization. This study addresses a significant information deficit by examining the temporal patterns of hospitalizations and emergency department visits related to individuals experiencing and getting out of homelessness.
4. Homeless individuals frequently incur elevated healthcare expenses due to restricted access to primary care, increased dependence on emergency services, and heightened hospitalization rates for preventable conditions, resulting in substantial financial burdens for taxpayers and healthcare systems. Factors contributing to elevated healthcare expenditures encompass restricted access to primary care, resulting in several homeless individuals lacking health insurance, a primary care physician, or consistent healthcare access, which culminates in deferred treatment and exacerbated health issues. This elucidates the augmented utilization of emergency departments, resulting in recurrent visits and elevated expenses per patient.
  Homeless individuals exhibit a higher propensity for hospitalization, frequently for ailments that may be addressed in more economical environments, hence exacerbating overall healthcare expenditures related to mental health and substance addiction. Housing instability can aggravate health issues and result in increased healthcare consumption. A 2006 study revealed that the mean annual expenditure for a recurrent emergency department client facing homelessness was USD 64,000. Hospital expenses for homeless patients, both in total and per hospitalization, can be markedly greater than those for housed patients [72].
  Individuals experiencing housing instability are more prone to possess Medicaid coverage or lack insurance altogether, resulting in their care expenses frequently being borne by state programs and hospitals. In addition to direct healthcare expenses, homelessness incurs secondary economic consequences, including diminished productivity and heightened expenditures related to incarceration.

Our analysis of SDOHs among cancer patients illuminates the societal challenges in the United States. Positioning societal problems focuses on the influence of social and environmental factors, together with their interaction with human traits, in sustaining racial and ethnic inequities in healthcare access and outcomes. In numerous practical applications, researchers investigate the extent to which community and environmental factors affect racial and ethnic differences in outcomes. In the United States, individuals facing homelessness may encounter risk factors arising from societal structural issues, including experiences in foster care, physical abuse, incarceration, suicide attempts, and psychiatric disorders, including addiction [73]. The impact of foster care experience on homelessness demonstrates the numerous interconnected societal structural elements. Regardless of the educational alternatives available to them, kids may encounter further maltreatment and instability. Furthermore, they shift to independent living with constrained means and limited access to services [74]. These structural risk factors collectively render individuals with foster care experience and anyone impacted by these factors susceptible to homelessness [75]. Significantly, from this viewpoint, a distinction exists between the consequences of residing in a low-income area and those associated with individual poverty, as evidenced by the two cohorts of social determinants of health we analyzed—homelessness and living alone. This article views SDOHs—homelessness and solitary living—not as a population-level predictor of outcomes but as a social phenomenon.

## 5. Conclusions

This study demonstrates that SDOHs correlate with poorer outcomes and inequities among cancer patients. This observation should justify additional inquiry. The severity and impact of inadequate social determinants of health are typically more pronounced for cancer patients, making it essential to create multimodal techniques to evaluate their unmet needs. Consequently, our inquiry into this domain elucidates the potential use of machine learning analytics for future applications in anticipating unfavorable events, care patterns, or outcomes that may be extensively deliberated in forthcoming healthcare contexts.

## 6. Limitations

This study’s findings should be interpreted in light of some limitations. First, the quality of data input is largely influenced by and is subject to variance among the individuals who provided the information. However, the volume of information should significantly reduce the possibility of significant inconsistencies. Second, we were not able to completely cover all aspects of SDOHs, which is beyond the scope of this study. Third, although highly unusual, it is possible for a patient to be counted more than once if readmitted after being discharged from the hospital because NIS statistics include discharges. Fourth, this cross-sectional study is incapable of inferring causality due to the inability to create a chronological sequence. Nonetheless, our analysis is employed to produce descriptive data concerning the outcome load in cancer patients or to ascertain background exposure rates. Consequently, our analysis in this domain elucidates the potential link, suggesting a correlation rather than causation. Finally, when calculating the true cost of resource utilization, we were only able to use hospital charges. Although we attempted to account for potential confounding factors in the study, it is possible that some variables related to exposure status were left out of our investigation because of limitations in the dataset, in addition to self-reported opioid dependence and homelessness. In our forthcoming investigations, we will examine the conditions of homelessness across diverse communities and the correlation between individual variables, homelessness, and cancer within the framework of community disparities.

## Figures and Tables

**Table 1:** Characteristics of metastatic cancer patients reported with homelessness.

	Metastatic Cancer Patients Reported with Homelessness
n	2635
AGE (mean (SD))	56.83 (9.94)
Sex (%)	
Female	695 (26.4)
RACE (%)	
White	1560.0 (60.5)
Black	665.0 (25.8)
Hispanic	165.0 (6.4)
Asian and others	190.0 (7.4)
Median household income (based on current year)	
0–25th percentile	945.0 (46.2)
26th to 50th percentile	400.0 (19.6)
51st to 75th percentile	380.0 (18.6)
76th to 100th percentile	320.0 (15.6)
Expected primary payer (%)	
Medicare	745.0 (28.3)
Medicaid	1495.0 (56.7)
Private insurance	150.0 (5.7)
Self-pay, no charge, and other	245.0 (9.3)
Patient Location: NCHS Urban–Rural Code (%)	
“Central” counties of metro areas of ≥1 million population	1000.0 (46.3)
“Fringe” counties of metro areas of ≥1 million population	400.0 (18.5)
Counties in metro areas of 250,000–999,999 population.	515.0 (23.8)
Counties in metro areas of 50,000–249,999 population.	110.0 (5.1)
Micropolitan counties and non-metropolitan or micropolitan counties	135.0 (6.3)
Indicator of a transfer out of the hospital	
Transferred in	330.0 (12.6)
Elective admission (%)	510.0 (52.3)
Weekend admission (%)	645.0 (24.5)
Emergency admission (%)	2040.0 (77.4)
Weighted Elixhauser score (mean (SD))	22.25 (8.47)

Abbreviations: SD, standard deviation; NCHS, National Center for Health Statistics; Note: All frequencies and percentages are weighted.

**Table 2:** Baseline characteristics of prostate cancer patients with homelessness and PrbLA.

	Prostate Cancer Patients Without Homelessness (Weighted)	Prostate Cancer Patients with Homelessness (Weighted)	*p*-Value	Prostate Cancer Patients Without PrbLA (Weighted)	Prostate Cancer Patients with PrbLA (Weighted)	*p*-Value
n	208,985	425		209,055	355	
AGE (mean [SD])	71.5 (10.81)	64.6 (8.55)	<0.001	71.5 (10.81)	76.1 (10.20)	<0.001
Age groups (%)			<0.001			0.07
45–54	10,110 (5.1)	35 (8.9)				
55–64	44,790 (22.5)	170 (43.0)		44,900 (22.6)	60 (17.1)	
>65	143,919 (72.4)	190 (48.1)		143,820 (72.3)	290 (82.9)	
RACE (%)			<0.001			0.56
White	140,409 (69.8)	205 (50.0)		140,375 (69.7)	240 (69.6)	
Black	35,530 (17.7)	160 (39.0)		35,615 (17.7)	75 (21.7)	
Hispanic	14,250 (7.1)	20 (4.9)		14,260 (7.1)		
Other	11,000 (5.5)	25 (6.1)		11,005 (5.5)	20 (5.8)	
Expected primary payer (%)			<0.001			0.003
Medicare	13,7394 (65.8)	245 (57.6)		137,334 (65.8)	305 (85.9)	
Medicaid	9305 (4.5)	125 (29.4)		9415 (4.5)	15 (4.2)	
Private insurance	54,095 (25.9)	30 (7.1)		54,100 (25.9)	25 (7.0)	
Self-pay, no charge, and other	7855 (3.8)	25 (5.9)		7870 (3.8)		
Median household income (based on current year)			<0.001			0.28
0–25th percentile	52,215 (25.4)	190 (58.5)		52,290 (25.4)	115 (32.9)	
26th to 50th percentile	51,980 (25.3)	65 (20.0)		51,945 (25.3)	100 (28.6)	
51st to 75th percentile	50,665 (24.6)	45 (13.8)		50,630 (24.6)	80 (22.9)	
76th to 100th percentile	50,730 (24.7)	25 (7.7)		50,700 (24.7)	55 (15.7)	
Patient Location: NCHS Urban–Rural Code (%)			<0.001			0.31
“Central” counties of metro areas of ≥1 million population	61,895 (29.7)	185 (53.6)		61,975 (29.8)	105 (29.6)	
“Fringe” counties of metro areas of ≥1 million population	53,240 (25.6)	65 (18.8)		53,250 (25.6)	55 (15.5)	
Counties in metro areas of 250,000–999,999 population.	40,360 (19.4)	60 (17.4)		40,325 (19.4)	95 (26.8)	
Counties in metro areas of 50,000–249,999 population	19,265 (9.2)	25 (7.2)		19,250 (9.2)	40 (11.3)	
Micropolitan counties and non-metropolitan or micropolitan counties	33,565 (16.1)			33,515 (16.1)	60 (16.9)	
Admission type (%)			<0.001			<0.001
Elective	83,355 (40.0)	55 (12.9)		83,375 (40.0)	35 (9.9)	
Indicator of a transfer out of the hospital			0.01			<0.001
Transferred out	39,385 (18.9)	125 (29.8)		39,380 (18.9)	130 (36.6)	
Weighted Elixhauser score mean (SD))	14.38 (10.93)	13.62 (10.54)	0.513	14.37 (10.93)	17.8 (11.15)	0.006
Length of stay (geometric mean)	2.5 days	4.7 days	<0.001	2.5 days	4.1 days	<0.001
Total charge (geometric mean)	USD 41,476	USD 46,340	0.7	USD 41,476	USD 31,652	0.02
In-hospital mortality (%)	6895 (3.3)			6885 (3.3)	15 (4.2)	0.66
Anxiety (%)	14,345 (6.9)	100 (23.5)	<0.001	14,390 (6.9)	55 (15.5)	0.005
Depression (%)	16,550.0 (7.9)	95.0 (22.4)	<0.001	16,585 (7.9)	60 (16.9)	0.003
Anxiety and depression (%)	25,885.0 (12.4)	155.0 (36.5)	<0.001	25,940 (12.4)	100 (28.2)	<0.001
Emergency admission (%)	102,535 (49.1)	310.0 (72.9)	<0.001	102,625 (49.1)	220 (62.0)	0.05
Opioid abuse (%)	1320.0 (0.6)	20.0 (4.7)	<0.001			
Opioid long-term use (%)	2385.0 (1.1)	15.0 (3.5)	0.04	3905 (1.9)	20 (5.6)	0.01

Abbreviations: SD, standard deviation; NCHS, National Center for Health Statistics; USD, United States dollar; PrbLA, problems related to living alone. Note: All frequencies and percentages are weighted.

**Table 3:** Weighted generalized linear models estimating association between homelessness and the outcomes: anxiety and depression, LOS, and PCa; 2017 NIS (weighted n, 209,410).

	aOR (95% CI)		Coefficient and 95% CIs (Back Transformed from Log Transformation)
	Anxiety and Depression		LOS
PCa homelessness status Non-homelessness Homelessness	Reference 5.14 (3.17–8.35)		Reference 1.96 (1.03–3.74)
Age	0.99 (0.98–0.99)		1.04 (1.00–1.01)
RACE (%)			
White	Reference		Reference
Black	0.57 (0.51–0.63)		1.22 (1.14–1.30)
Hispanic	0.62 (0.54–0.72)		1.07 (0.97–1.18)
Asian and Native American and other	0.65 (0.53–0.79)		1.06 (0.95–1.18)
Expected primary payer			
Medicare	Reference		Reference
Medicaid	0.99 (0.85–1.15)		1.23 (1.09–1.39)
Private insurance	0.64 (0.58–0.70)		0.87 (0.82–0.92)
Self-pay and no charge and other	0.81 (0.68–0.98)		0.76 (0.64–0.89)
Patient Location: NCHS Urban–Rural Code			
Central counties of metro areas of ≥1 million population	Reference		Reference
“Fringe” counties of metro areas of ≥1 million population	0.94 (0.86–1.03)		0.97 (0.97–1.12)
Counties in metro areas of 250,000–999,999 population.	0.94 (0.85–1.04)		0.96 (0.98–1.13)
Counties in metro areas of 50,000–249,999 population.	0.89 (0.79–1.02)		0.73 (0.98–1.16)
Micropolitan counties and non-metropolitan or micropolitan counties	0.85 (0.76–0.94)		0.71 (0.88–1.05)
Elixhauser comorbidity score	0.99 (0.99–1.00)		1.07 (1.04–1.05)
Median household income			
0–25th percentile	Reference		Reference
26th to 50th percentile	0.97 (0.89–1.07)		0.93 (0.68–1.28)
51st to 75th percentile	0.98 (0.89–1.08)		1.15 (0.83–1.58)
76th to 100th percentile	0.95 (0.86–1.06)		0.85 (0.59–1.24)
Indicator of a transfer out of the hospital Not transferred out			Reference
Transferred out			2.20 (2.07–2.34)

Abbreviations: NIS, National Inpatient Sample; NCHS, National Center for Health Statistics; CI, confidence interval; aOR, adjusted odds ratio; PCa, prostate cancer; LOS, in-hospital length of stay.

**Table 4:** Baseline characteristics of breast cancer patients with and without homelessness and PrbLA.

	Breast Cancer Patients Without Homelessness (Weighted)	Breast Cancer Patients with Homelessness (Weighted)	*p*-Value	Breast Cancer Patients Without PrbLA (Weighted)	Breast Cancer Patients with PrbLA (Weighted)	*p*-Value
n	116,760	320		116,855	225	
AGE (mean [SD])	61.9 (14.19)	54.7 (11.42)	<0.001	61.8 (14.19)	70.8 (10.16)	<0.001
Age groups (%)			0.008			0.008
45–54	21,475.0 (22.3)	90.0 (40.0)		21,550.0 (22.4)	15.0 (7.0)	
55–64	26,995.0 (28.0)	70.0 (31.1)		27,020.0 (28.1)	45.0 (20.9)	
>65	47,790.0 (49.6)	65.0 (28.9)		47,700.0 (49.5)	155.0 (72.1)	
RACE (%)			0.07			
White	74,400 (65.5)	170.0 (53.1)		74,401.0(65.5)	165.0 (76.7)	
Black	19,630.0 (17.3)	95.0 (29.7)		19,690.0 (17.3)	35.0 (16.3)	
Hispanic	10,280.0 (9.1)	20.0 (6.2)		10,295.0 (9.1)		
Other	9260.0 (8.2)	35.0 (10.9)		9285.0 (8.2)	10.0 (4.7)	
Expected primary payer (%)						
Medicare	52,950.0 (45.4)	110.0 (34.9)		52,895.0 (45.3)	165.0 (73.3)	
Medicaid	15,620.0 (13.4)	170.0 (54.0)		15,770.0 (13.5)	20.0 (8.9)	
Private insurance	43,685.0 (37.5)			43,655.0 (37.4)	40.0 (17.8)	
Self-pay, no charge, and other	4350.0 (3.7)	25.0 (7.9)		4375.0 (3.7)		
Median household income (based on current year)			<0.001			0.44
0–25th percentile	29,230.0 (25.4)	130.0 (52.0)		29,310.0 (25.5)	50.0 (22.7)	
26th to 50th percentile	27,325.0 (23.7)	50.0 (20.0)		27,340.0 (23.8)	35.0 (15.9)	
51st to 75th percentile	28,225.0 (24.5)	50.0 (20.0)		28,220.0 (24.5)	55.0 (25.0)	
76th to 100th percentile	30,275.0 (26.3)	20.0 (8.0)		30,215.0 (26.3)	80.0 (36.4)	
Patient Location: NCHS Urban–Rural Code (%)						0.36
“Central” counties of metro areas of ≥1 million population	38,900.0 (33.4)	105.0 (41.2)		38,945.0 (33.4)	60.0 (26.7)	
“Fringe” counties of metro areas of ≥1 million population	31,770.0 (27.3)	80.0 (31.4)		31,805.0 (27.3)	45.0 (20.0)	
Counties in metro areas of 250,000–999,999 population.	21,715.0 (18.6)	55.0 (21.6)		21,725.0 (18.6)	45.0 (20.0)	
Counties in metro areas of 50,000–249,999 population	8545.0 (7.3)			8520.0 (7.3)	25.0 (11.1)	
Micropolitan counties and non-metropolitan or micropolitan counties	15,539.9 (13.3)	15.0 (5.9)		15,504.9 (13.3)	50.0 (22.2)	
Admission type (%)			<0.001			0.46
Elective	42,520.0 (36.5)	25.0 (7.8)		42,475.0 (36.4)	70.0 (31.1)	
Indicator of a transfer out of the hospital			<0.001			<0.001
Transferred out	16,385.0 (14.0)	115.0 (35.9)		16,430.0 (14.1)	70.0 (31.1)	
Weighted Elixhauser score mean (SD))	13.32 (10.20)	13.67 (9.90)	0.79	13.32 (10.20)	14.89 (10.21)	0.27
Length of stay (geometric mean)	2.6 days	4.1 days	0.2	2.6 days	4.9 days	<0.001
Total charge (geometric mean)	USD 40,622	USD 48,644	0.2	USD 40,622	USD 34,636	0.18
In-hospital mortality (%)	3770.0 (3.2)	15.0 (4.7)	0.5	3780.0 (3.2)		
Anxiety (%)	18,505.0 (15.8)	90.0 (28.1)	0.009	18,555.0 (15.9)	40.0 (17.8)	0.74
Depression (%)	16,750.0 (14.3)	55.0 (17.2)	0.538	16,730.0 (14.3)	75.0 (33.3)	<0.001
Anxiety and depression (%)	27,815.0 (23.8)	115.0 (35.9)	0.04	27,845.0 (23.8)	85.0 (37.8)	0.03
Emergency admission (%)	58,760 (50.3)	265.0 (82.8)	<0.001	58,899.9 (50.4)	125.0 (55.6)	0.54
Opioid abuse (%)	825.0 (0.7)	25.0 (7.8)	<0.001			
Opioid long-term use (%)				3000.0 (2.6)	20.0 (8.9)	0.008

Abbreviations: SD, standard deviation; NCHS, National Center for Health Statistics; USD, United States dollar; PrbLA, problems related to living alone. Note: All frequencies and percentages are weighted.

**Table 5:** Weighted generalized linear models estimating the association between homelessness and the outcomes: anxiety and breast cancer; 2017 NIS (weighted n, 117,080).

	aOR (95% CI)
	Anxiety
Breast cancer homelessness status Non-homelessness Homelessness	Reference 2.07 (1.06–4.03)
Age	0.97 (0.97–0.98)
RACE (%)	
White	Reference
Black	0.47 (0.42–0.54)
Hispanic	0.56 (0.48–0.66)
Asian and Native American and other	0.44 (0.34–0.56)
Expected primary payer	
Medicare	Reference
Medicaid	0.94 (0.81–1.09)
Private insurance	0.76 (0.68–0.85)
Self-pay and no charge and other	0.71 (0.57–0.88)
Patient Location: NCHS Urban–Rural Code	
Central counties of metro areas of ≥1 million population	Reference
Fringe” counties of metro areas of ≥1 million population	1.15 (1.04–1.28)
Counties in metro areas of 250,000–999,999 population.	1.10 (0.97–1.25)
Counties in metro areas of 50,000–249,999 population.	1.05 (0.89–1.24)
Micropolitan counties and non-metropolitan or micropolitan counties	0.99 (0.87–1.14)
Elixhauser comorbidity score	0.99 (0.99–1.00)
Median household income	
0–25th percentile	Reference
26th to 50th percentile	0.93 (0.84–1.04)
51st to 75th percentile	0.94 (0.84–1.05)
76th to 100th percentile	0.95 (0.85–1.08)

Abbreviations: NIS, National Inpatient Sample; NCHS, National Center for Health Statistics; CI, confidence interval; aOR, adjusted odds ratio; LOS, in-hospital length of stay.

**Table 6:** Baseline characteristics of lung cancer patients with homelessness and PrbLA.

	Lung Cancer Patients Without Homelessness (Weighted)	Lung Cancer Patients with Homelessness (Weighted)	*p*-Value	Lung Cancer Patients Without PrbLA (Weighted)	Lung Cancer Patients with PrbLA (Weighted)	*p*-Value
n	401,665	1345		402,190	820	
AGE (mean [SD])	69.09 (10.54)	60.41 (8.27)	<0.001	69.06 (10.55)	72.22 (9.59)	<0.001
Age groups (%)			<0.001			0.04
45–54	27,065.0 (7.2)	255.0 (20.4)		27,290.0 (7.2)	30.0 (3.8)	
55–64	92,695.0 (24.6)	675.0 (54.0)		93,220.0 (24.7)	150.0 (19.2)	
>65	25,7625.0 (68.3)	320.0 (25.6)		257,345.0 (68.1)	600.0 (76.9)	
Sex (%)			<0.001			0.005
Female	196,665.0 (49.0)	265.0 (19.7)		196,445.0 (48.8)	485.0 (59.1)	
Depression (%)	51,775.0 (12.9)	210.0 (15.6)	0.22	51,840.0 (12.9)	145.0 (17.7)	0.07
Anxiety (%)	64,185.0 (16.0)	265.0 (19.7)	0.09	64,275.0 (16.0)	175.0 (21.3)	0.07
Anxiety and depression (%)	93,070.0 (23.2)	400.0 (29.7)	<0.001			0.88
Emergency admission (%)	263,760.0 (65.7)	1030.0 (76.6)	0.88	264,245.0 (65.7)	545.0 (66.5)	0.88
Opioid abuse (%)	4955.0 (1.2)	80.0 (5.9)	<0.001	5020.0 (1.2)	15.0 (1.8)	0.59
Opioid long-term use (%)	12,450.0 (3.1)	50.0 (3.7)	0.56	12,470.0 (3.1)	30.0 (3.7)	0.68
RACE (%)			<0.001			0.53
White	303,165.0 (77.6)	855.0 (65.0)		303,370.0 (77.5)	650.0 (82.3)	
Black	48,210.0 (12.3)	310.0 (23.6)		48,435.0 (12.4)	85.0 (10.8)	
Hispanic	17,900.0 (4.6)	70.0 (5.3)		17,950.0 (4.6)	20.0 (2.5)	
Other	21,510.0 (5.5)	80.0 (6.1)		21,555.0 (5.5)	35.0 (4.4)	
Expected primary payer (%)			<0.001			0.015
Medicare	267,405.0 (66.7)	425.0 (31.6)		267,190.0 (66.5)	640.0 (78.0)	
Medicaid	39,045.0 (9.7)	760.0 (56.5)		39,755.0 (9.9)	50.0 (6.1)	
Private insurance	775,25.0 (19.3)	60.0 (4.5)		77,480.0 (19.3)	105.0 (12.8)	
Self-pay, no charge, and other	17,160.0 (4.3)	100.0 (7.4)		17,235.0 (4.3)	25.0 (3.0)	
Median household income (based on current year)			<0.001			0.537
0–25th percentile	119,805.1 (30.2)	530.0 (48.6)		120,130.1 (30.3)	205.0 (25.5)	
26th to 50th percentile	108,425.0 (27.4)	185.0 (17.0)		108,370.0 (27.3)	240.0 (29.8)	
51st to 75th percentile	91,905.0 (23.2)	215.0 (19.7)		91,910.0 (23.2)	210.0 (26.1)	
76th to 100th percentile	76,109.9 (19.2)	160.0 (14.7)		76,119.9 (19.2)	150.0 (18.6)	
Patient Location: NCHS Urban–Rural Code (%)			<0.001			0.472
“Central” counties of metro areas of ≥1 million population	103,600.0 (25.8)	440.0 (38.8)		103,805.0 (25.9)	235.0 (28.7)	
“Fringe” counties of metro areas of ≥1 million population	101,795.0 (25.4)	240.0 (21.1)		101,830.0 (25.4)	205.0 (25.0)	
Counties in metro areas of 250,000–999,999 population.	80,565.0 (20.1)	320.0 (28.2)		807,55.0 (20.1)	130.0 (15.9)	
Counties in metro areas of 50,000–249,999 population	40,145.0 (10.0)	85.0 (7.5)		401,10.0 (10.0)	120.0 (14.6)	
Micropolitan counties and non-metropolitan or micropolitan counties	74,875.0 (18.7)	50.0 (4.4)		747,95.0 (18.6)	130.0 (15.9)	
Admission type (%)			<0.001			0.15
Elective	75,095.0 (18.7)	125.0 (9.3)		75,105.0 (18.7)	115.0 (14.0)	
Indicator of a transfer out of the hospital			<0.001			0.04
Transferred out	86,370.0 (21.5)	475.0 (35.3)		86,605.0 (21.6)	240.0 (29.3)	
Weighted Elixhauser score mean (SD))	19.10 (9.97)	18.19 (9.79)	0.14	19.10 (9.97)	18.99 (9.51)	0.88
Length of stay (geometric mean)	3.8 days	6.2 days	<0.001	3.8 days	4.4 days	0.09
Total charge (geometric mean)	USD 42,055	USD 58,251	<0.001	USD 42,055	USD 41,190	0.81
In-hospital mortality (%)	34,960.0 (8.7)	110.0 (8.2)	0.76	35,030.0 (8.7)	40.0 (4.9)	0.07
Depression (%)	51,775.0 (12.9)	210.0 (15.6)	0.22	51,840.0 (12.9)	145.0 (17.7)	0.07
Anxiety (%)	64,185.0 (16.0)	265.0 (19.7)	0.09	64,275.0 (16.0)	175.0 (21.3)	0.07
Anxiety and depression (%)	93,070.0 (23.2)	400.0 (29.7)	<0.001			0.88
Emergency admission (%)	263,760.0 (65.7)	1030.0 (76.6)	0.88	264,245.0 (65.7)	545.0 (66.5)	0.88
Opioid abuse (%)	4955.0 (1.2)	80.0 (5.9)	<0.001	5020.0 (1.2)	15.0 (1.8)	0.59
Opioid long-term use (%)	12,450.0 (3.1)	50.0 (3.7)	0.56	12,470.0 (3.1)	30.0 (3.7)	0.68

Abbreviations: SD, standard deviation; NCHS, National Center for Health Statistics; USD, United States dollar; PrbLA, problems related to living alone. Note: All frequencies and percentages are weighted.

**Table 7:** Weighted generalized linear models estimating the association between homelessness and the outcomes: anxiety and depression, LOS, and lung cancer; 2017 NIS (weighted n, 403,010).

	aOR (95% CI)	Coefficient and 95% CIs (Back Transformed from Log Transformation)
	Anxiety and Depression	LOS
Lung cancer homelessness status Non-homelessness Homelessness	Reference 1.38 (1.02–1.85)	Reference 1.84 (1.40–2.42)
Age	0.97 (0.97–0.97)	0.99 (0.99–0.99)
RACE (%)		
White	Reference	Reference
Black	0.51 (0.48–0.55)	1.22 (1.05–1.17)
Hispanic	0.66 (0.60–0.72)	1.07 (0.93–1.09)
Asian and Native American and other	0.53 (0.47–0.59)	1.13 (1.04–1.23)
Expected primary payer		
Medicare	Reference	Reference
Medicaid	0.91 (0.85–0.97)	0.91 (0.85–0.97)
Private insurance	0.78 (0.74–0.83)	0.89 (0.84–0.93)
Self-pay and no charge and other	0.65 (0.59–0.72)	0.67 (0.59–0.75)
Patient Location: NCHS Urban–Rural Code		
Central counties of metro areas of ≥1 million population	Reference	Reference
Fringe” counties of metro areas of ≥1 million population	1.03 (0.96–1.09)	0.97 (0.92–1.02)
Counties in metro areas of 250,000–999,999 population.	1.04 (0.96–1.12)	1.00 (0.95–1.06)
Counties in metro areas of 50,000–249,999 population.	1.03 (0.95–1.13)	1.00 (0.94–1.08)
Micropolitan counties and non-metropolitan or micropolitan counties	0.92 (0.85–0.99)	0.88 (0.83–0.94)
Elixhauser comorbidity score	0.98 (0.98–0.99)	1.02 (1.02–1.03)
Median household income		
0–25th percentile	Reference	Reference
26th to 50th percentile	0.99 (0.95–1.05)	0.95 (0.90–0.99)
51st to 75th percentile	1.01 (0.96–1.07)	0.94 (0.89–0.99)
76th to 100th percentile	0.99 (0.93–1.07)	0.93 (0.87–0.98)
Indicator of a transfer out of the hospital Non-transferred out		Reference
Transferred out		2.20 (2.07–2.34)

Abbreviations: NIS, National Inpatient Sample; NCHS, National Center for Health Statistics; CI, confidence interval; aOR, adjusted odds ratio; LOS, in-hospital length of stay.

**Table 8:** Baseline characteristics of cancers of the lip, oral cavity, and pharynx (CLOP) patients with homelessness and PrbLA.

	CLOP Patients Without Homelessness (Weighted)	CLOP Patients with Homelessness (Weighted)	*p*-Value
n	53,890	375	
AGE (mean [SD])	64.06 (12.22)	54.85 (8.53)	<0.001
Female (%)			0.001
	15,540.0 (28.8)	45.0 (12.0)	
Age groups (%)			<0.001
45–54	7700.0 (16.1)	130.0 (41.3)	
55–64	15,970.0 (33.4)	165.0 (52.4)	
>65	24,105.0 (50.5)	20.0 (6.3)	
RACE (%)			0.09
White	38,965.0 (74.9)	245.0 (67.1)	
Black	5500.0 (10.6)	70.0 (19.2)	
Hispanic	3360.0 (6.5)	30.0 (8.2)	
Other	4220.0 (8.1)	20.0 (5.5)	
Expected primary payer (%)			<0.001
Medicare	27,195.0 (50.6)	85.0 (22.7)	
Medicaid	8130.0 (15.1)	220.0 (58.7)	
Private insurance	15,540.0 (28.9)	25.0 (6.7)	
Self-pay, no charge, and other	2930.0 (5.4)	45.0 (12.0)	
Median household income (based on current year)			0.415
0–25th percentile	14,990.0 (28.3)	115.0 (37.7)	
26th to 50th percentile	13,965.0 (26.4)	75.0 (24.6)	
51st to 75th percentile	12,725.0 (24.0)	60.0 (19.7)	
76th to 100th percentile	11,255.0 (21.3)	55.0 (18.0)	
Patient Location: NCHS Urban–Rural Code (%)			0.032
“Central” counties of metro areas of ≥1 million population	15,485.0 (28.8)	115.0 (37.1)	
“Fringe” counties of metro areas of ≥1 million population	13,835.0 (25.7)	55.0 (17.7)	
Counties in metro areas of 250,000–999,999 population.	10,960.0 (20.4)	95.0 (30.6)	
Counties in metro areas of 50,000–249,999 population	5150.0 (9.6)	30.0 (9.7)	
Micropolitan counties and non-metropolitan or micropolitan counties	8315.0 (15.5)	15.0 (4.8)	
Admission type (%)			<0.001
Elective	19,600.0 (36.5)	45.0 (12.0)	
Indicator of a transfer out of the hospital			0.002
Transferred out	10,335.0 (19.2)	125.0 (33.3)	
Weighted Elixhauser score mean (SD)	15.71 (9.46)	14.59 (10.76)	0.370
Length of stay (geometric mean)	3.7 days	8.01 days	<0.001
Total charge (geometric mean)	USD 48,983	USD 57,449	0.2
Anxiety (%)	7380.0 (13.7)	105.0 (28.0)	<0.001
Depression (%)	6515.0 (12.1)	80.0 (21.3)	0.02
Anxiety and depression (%)	11,140.0 (20.7)	145.0 (38.7)	<0.001
Emergency admission (%)	26,285.0 (48.8)	245.0 (65.3)	<0.001

Abbreviations: SD, standard deviation; NCHS, National Center for Health Statistics; USD, United States dollar; PrbLA, problems related to living alone. Note: All frequencies and percentages are weighted.

## Data Availability

Data is unavailable due to privacy or ethical restrictions as per the requirements of HCUP of the AHRQ.

## Supplementary Materials

The following supporting information can be downloaded at: https://www.mdpi.com/article/10.3390/cancers17071121/s1, File: Supplementary file; Table S1: Weighted generalized linear models estimating association between PrbLA and the outcomes: anxiety and depression, and LOS, PCa 2017 NIS (weighted n = 209,410); Table S2: Weighted generalized linear models estimating association between PrbLA and the outcomes: anxiety and depression, and LOS, Breast Cancers 2017 NIS (weighted n = 117,080); Table S3: Weighted generalized linear models estimating association between PrbLA and the outcome: LOS, Lung patients stratified to Females, 2017 NIS (weighted n = 196,930); Table S4: Weighted generalized linear models estimating association between homelessness and the outcomes: anxiety and depression, and LOS, CLOP 2017 NIS (weighted n = 54,265).
