# Supplementary material for: Predictors for Emergency Admission Among Homeless Metastatic Cancer Patients and Association of Social Determinants of Health with Negative Health Outcomes
Source: Cancers (Basel). 2025 Mar 27;17(7):1121. doi: 10.3390/cancers17071121 (PMC11987736; doi:10.3390/cancers17071121)
Supplement: Supplementary file 1 [file cancers-17-01121-s001.zip › Supplementary file.pdf]

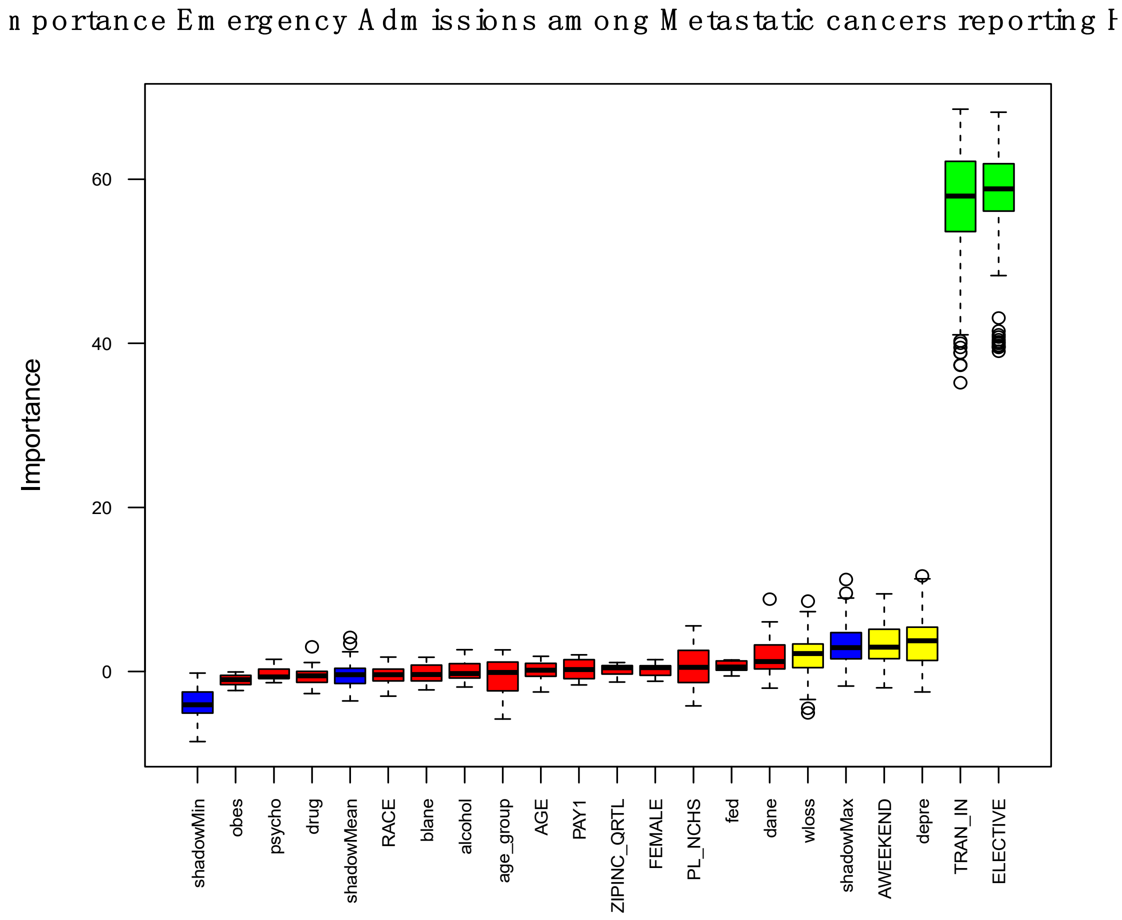

Supplementary Figure. Boruta result plot for emergency admissions among metastatic cancers. Blue boxplots correspond to minimal, average and maximum Z score of a shadow attribute. Red and green boxplots represent Z scores of respectively rejected and confirmed attributes.

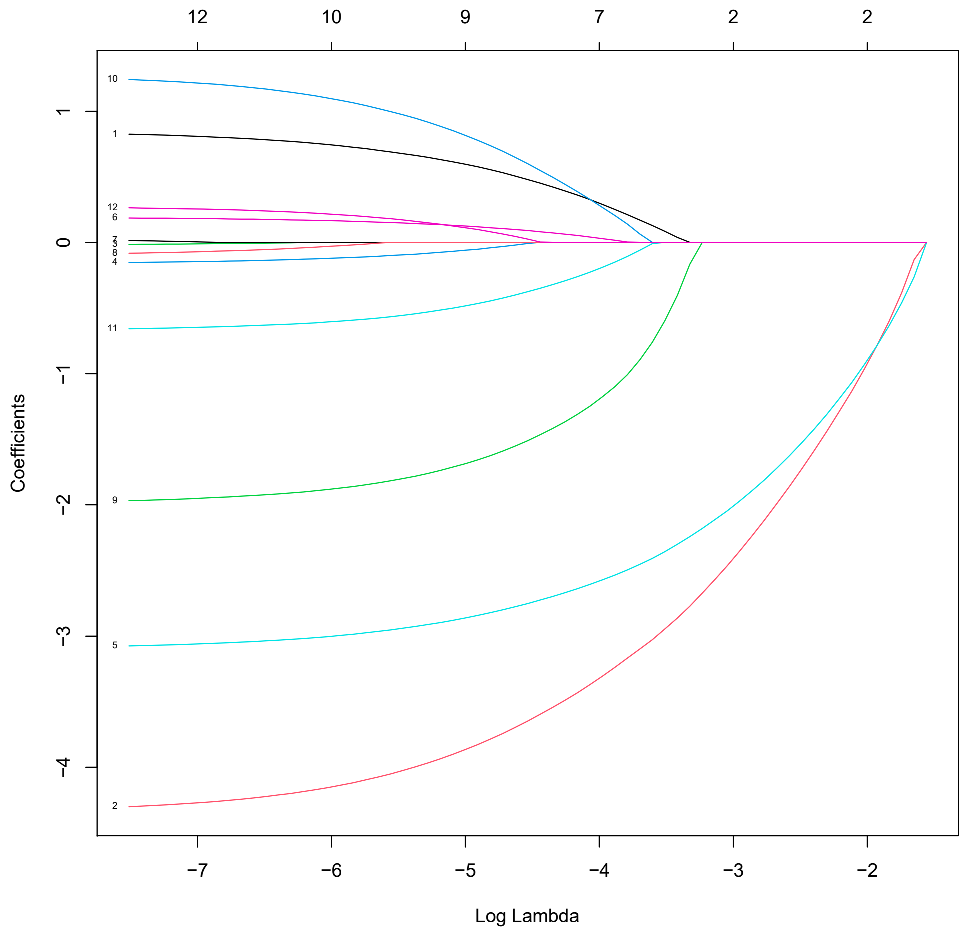

Supplementary figure. Plot of estimated coefficient of lambda for lasso (emergency admissions).

The plot indicates the number of predictors (variables) the model is using, from all predictors to sparser models. As lambda (λ) increase the predictors shrinks to 0.

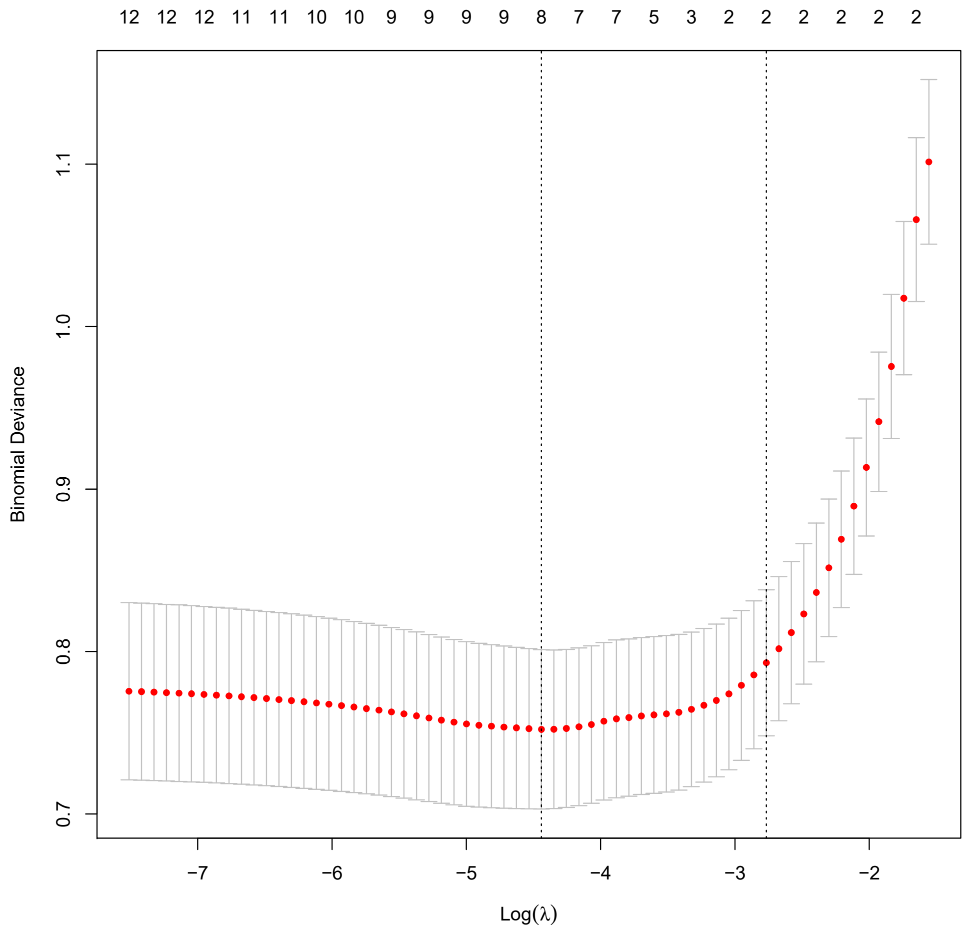

<u>Supplementary Figure shows cross-validation for lasso (Emergency admission</u>). In the plot, lambda demonstrate the tuning parameter: the 10-fold cross-validated binomial deviance as a function of (log) lambda (λ) for the lasso regularized model. This task helps tuning the parameter or assists in optimization of lasso with reference to choosing the best lambda

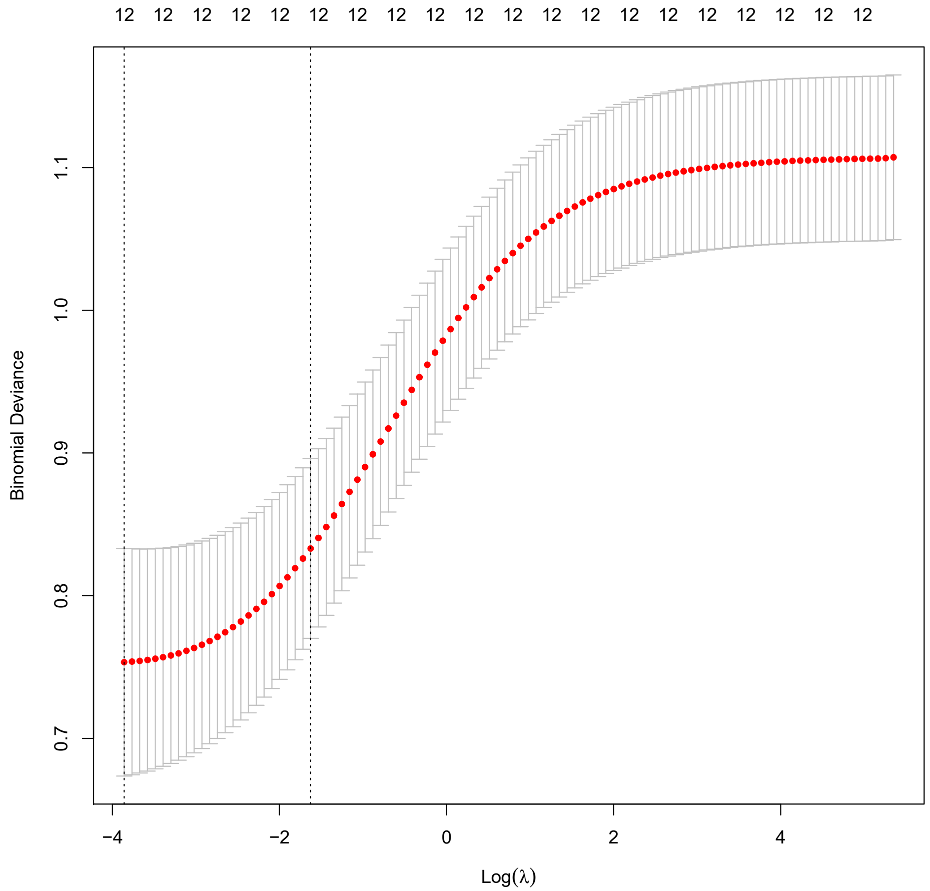

<u>Supplementary Figure</u> <u>shows cross-validation for ridge (</u>**<u>Emergency admission</u>**<u>).</u> In the plot, lambda demonstrate the tuning parameter: the 10-fold cross-validated binomial deviance as a function of (log) lambda (λ) for the lasso regularized model. This task helps tuning the parameter or assists in optimization of lasso with reference to choosing the best lambda

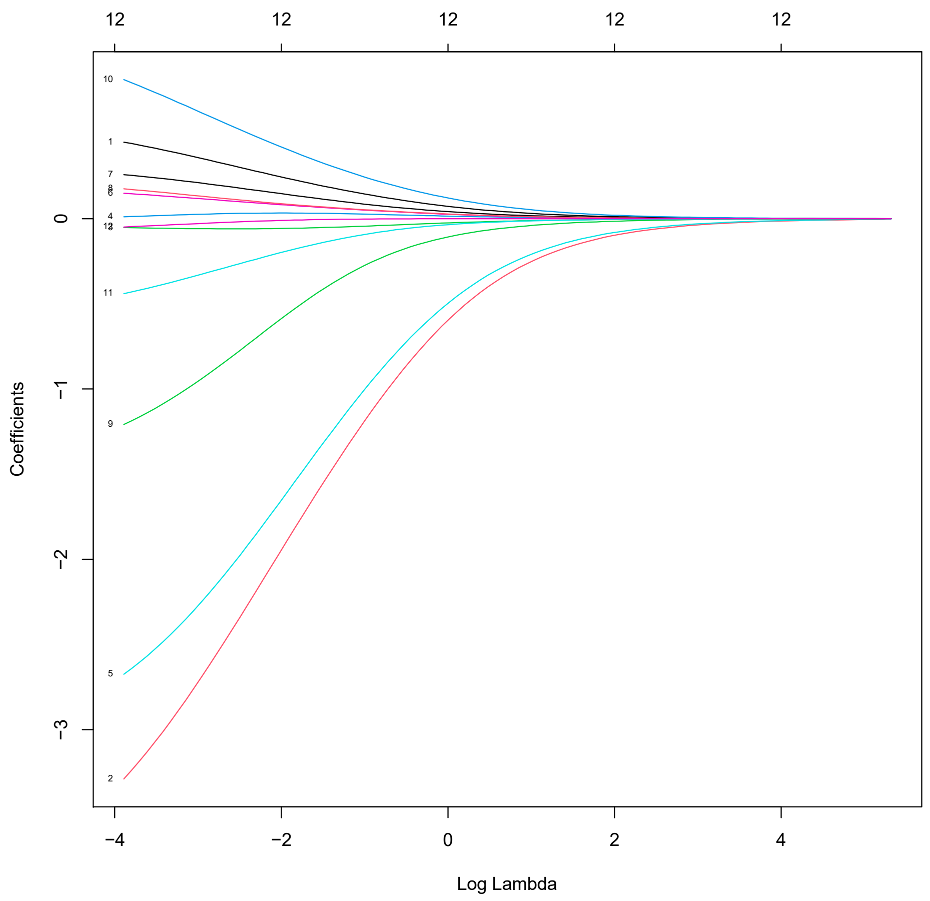

Supplementary figure. <u>Plot of estimated coefficient of lambda for ridge (Emergency admissions)</u>

The plot indicates the number of predictors (variables) the model is using, from all predictors to sparser models. As lambda (λ) increase the predictors shrinks to 0.

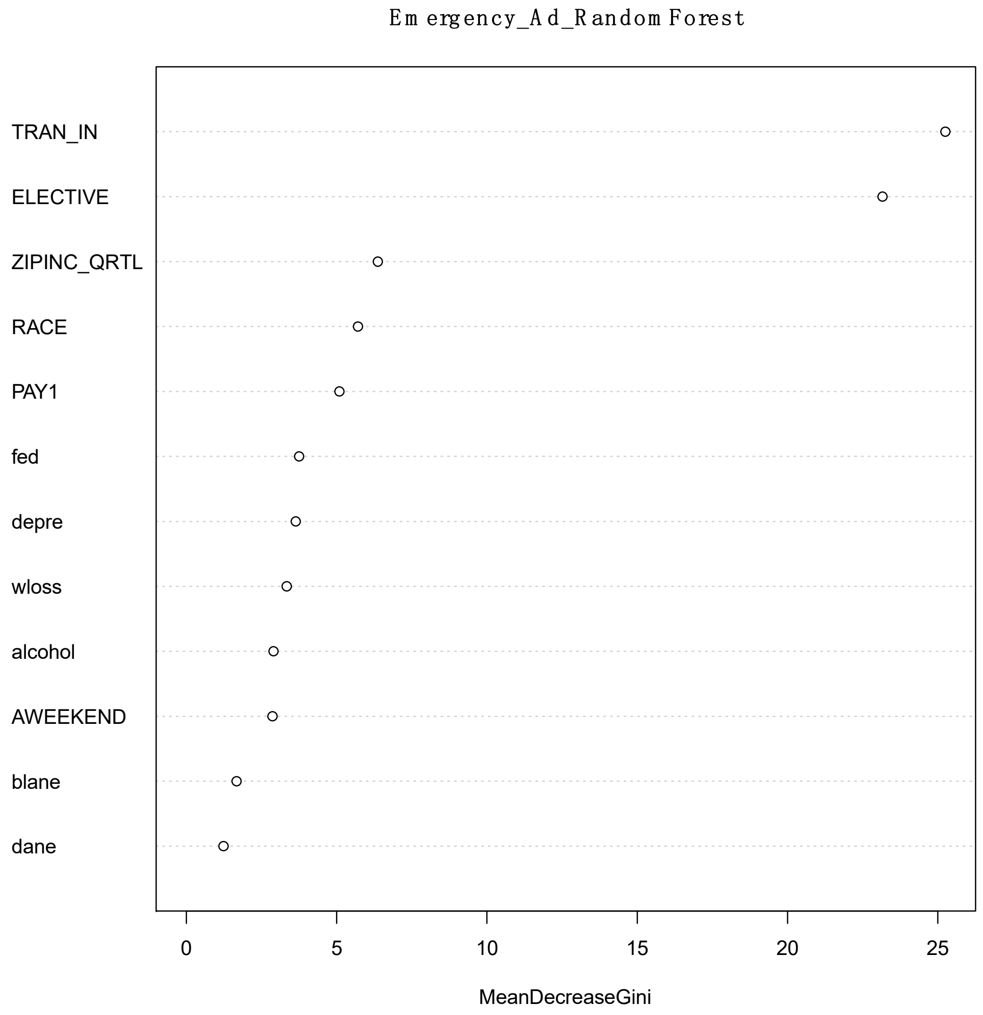

Summary plot. Random Forest Variable of importance for Emergency admission among metastatic cancers.

ICD 10 CM billable Codes.

(Lung Cancer).

- **C34.00 Malignant neoplasm of unspecified main bronchus**
- C34.01 Malignant neoplasm of right main bronchus
- C34.02 Malignant neoplasm of left main bronchus
- C34.10 Malignant neoplasm of upper lobe, unspecified bronchus or lung
- C34.11 Malignant neoplasm of upper lobe, right bronchus or lung
- C34.12 Malignant neoplasm of upper lobe, left bronchus or lung
- C34.2 Malignant neoplasm of middle lobe, bronchus or lung
- C34.30 Malignant neoplasm of lower lobe, unspecified bronchus or lung
- C34.31 Malignant neoplasm of lower lobe, right bronchus or lung
- C34.32 Malignant neoplasm of lower lobe, left bronchus or lung
- C34.80 Malignant neoplasm of overlapping sites of unspecified bronchus and lung
- C34.81 Malignant neoplasm of overlapping sites of right bronchus and lung
- C34.82 Malignant neoplasm of overlapping sites of left bronchus and lung
- C34.90 Malignant neoplasm of unspecified part of unspecified bronchus or lung
- C34.91 Malignant neoplasm of unspecified part of right bronchus or lung
- C34.92 Malignant neoplasm of unspecified part of left bronchus or lung

(Prostate)

C61 Malignant neoplasm of prostate

(Breast)

"C50011", Malignant neoplasm of nipple and areola, right female breast
"C50111", Malignant neoplasm of central portion, right female breast
"C50211", Malignant neoplasm of upper-inner quadrant, right female breast
"C50311", Malignant neoplasm of lower-inner quadrant, right female breast
"C50411", Malignant neoplasm of upper-outer quadrant, right female breast
"C50511", Malignant neoplasm of lower-outer quadrant, right female breast
"C50611", Malignant neoplasm of axillary tail, right female breast
"C50811", Malignant neoplasm of overlapping sites, right female breast
"C50911", Malignant neoplasm of unspecified site, right female breast

"D0501",	Lobular carcinoma in situ, right breast
"D0511",	Intraductal carcinoma in situ, right breast
"D0581",	Other specified type of carcinoma in situ, right breast

"C50012",	Malignant neoplasm of nipple and areola, left female breast
"C50112",	Malignant neoplasm of central portion, left female breast
"C50212",	Malignant neoplasm of upper-inner quadrant, left female breast
"C50312",	Malignant neoplasm of lower-inner quadrant, left female breast
"C50412",	Malignant neoplasm of upper-outer quadrant, left female breast
"C50512",	Malignant neoplasm of lower-outer quadrant, left female breast
"C50612",	Malignant neoplasm of axillary tail, left female breast
"C50812",	Malignant neoplasm of overlapping sites, left female breast
"C50912",	Malignant neoplasm of unspecified site, left female breast
"D0502",	Lobular carcinoma in situ, left breast
"D0512",	Intraductal carcinoma in situ, left breast
"D0582"	Other specified type of carcinoma in situ, left breast

ICD 10 billable Codes for Malignant neoplasms of lip, oral cavity and pharynx

(C00-C14).

- C00.0 - Malignant neoplasm of external upper lip **BILLABLE CODE**
- C00.1 - Malignant neoplasm of external lower lip **BILLABLE CODE**
- C00.2 - Malignant neoplasm of external lip, unspecified **BILLABLE CODE**
- C00.3 - Malignant neoplasm of upper lip, inner aspect **BILLABLE CODE**
- C00.4 - Malignant neoplasm of lower lip, inner aspect **BILLABLE CODE**
- C00.5 - Malignant neoplasm of lip, unspecified, inner aspect **BILLABLE CODE**
- C00.6 - Malignant neoplasm of commissure of lip, unspecified **BILLABLE CODE**
- C00.8 - Malignant neoplasm of overlapping sites of lip **BILLABLE CODE**
- C00.9 - Malignant neoplasm of lip, unspecified **BILLABLE CODE**

Malignant neoplasm of base of tongue (C01)

- C01 - Malignant neoplasm of base of tongue **BILLABLE CODE**

Malignant neoplasm of other and unspecified parts of tongue (C02)

- C02.0 - Malignant neoplasm of dorsal surface of tongue **BILLABLE CODE**
- C02.1 - Malignant neoplasm of border of tongue **BILLABLE CODE**
- C02.2 - Malignant neoplasm of ventral surface of tongue **BILLABLE CODE**
- C02.3 - Malig neoplasm of anterior two-thirds of tongue, part unsp **BILLABLE CODE**
- C02.4 - Malignant neoplasm of lingual tonsil **BILLABLE CODE**
- C02.8 - Malignant neoplasm of overlapping sites of tongue **BILLABLE CODE**
- C02.9 - Malignant neoplasm of tongue, unspecified **BILLABLE CODE**

Malignant neoplasm of gum (C03)

- C03.0 - Malignant neoplasm of upper gum **BILLABLE CODE**
- C03.1 - Malignant neoplasm of lower gum **BILLABLE CODE**
- C03.9 - Malignant neoplasm of gum, unspecified **BILLABLE CODE**

Malignant neoplasm of floor of mouth (C04)

- C04.0 - Malignant neoplasm of anterior floor of mouth **BILLABLE CODE**
- C04.1 - Malignant neoplasm of lateral floor of mouth **BILLABLE CODE**
- C04.8 - Malignant neoplasm of overlapping sites of floor of mouth **BILLABLE CODE**
- C04.9 - Malignant neoplasm of floor of mouth, unspecified **BILLABLE CODE**

Malignant neoplasm of palate (C05)

- C05.0 - Malignant neoplasm of hard palate **BILLABLE CODE**
- C05.1 - Malignant neoplasm of soft palate **BILLABLE CODE**
- C05.2 - Malignant neoplasm of uvula **BILLABLE CODE**

- C05.8 - Malignant neoplasm of overlapping sites of palate **BILLABLE CODE**
- C05.9 - Malignant neoplasm of palate, unspecified **BILLABLE CODE**

Malignant neoplasm of other and unspecified parts of mouth (C06)

- C06.0 - Malignant neoplasm of cheek mucosa **BILLABLE CODE**
- C06.1 - Malignant neoplasm of vestibule of mouth **BILLABLE CODE**
- C06.2 - Malignant neoplasm of retromolar area **BILLABLE CODE**
- C06.80 - Malignant neoplasm of ovrlp sites of unsp parts of mouth **BILLABLE CODE**
- C06.89 - Malignant neoplasm of overlapping sites of oth prt mouth **BILLABLE CODE**
- C06.9 - Malignant neoplasm of mouth, unspecified **BILLABLE CODE**

Malignant neoplasm of parotid gland (C07)

- C07 - Malignant neoplasm of parotid gland **BILLABLE CODE**

Malignant neoplasm of other and unsp major salivary glands (C08)

- C08.0 - Malignant neoplasm of submandibular gland **BILLABLE CODE**
- C08.1 - Malignant neoplasm of sublingual gland **BILLABLE CODE**
- C08.9 - Malignant neoplasm of major salivary gland, unspecified **BILLABLE CODE**

Malignant neoplasm of tonsil (C09)

- C09.0 - Malignant neoplasm of tonsillar fossa **BILLABLE CODE**
- C09.1 - Malig neoplasm of tonsillar pillar (anterior) (posterior) **BILLABLE CODE**
- C09.8 - Malignant neoplasm of overlapping sites of tonsil **BILLABLE CODE**
- C09.9 - Malignant neoplasm of tonsil, unspecified **BILLABLE CODE**

Malignant neoplasm of oropharynx (C10)

- C10.0 - Malignant neoplasm of vallecula **BILLABLE CODE**
- C10.1 - Malignant neoplasm of anterior surface of epiglottis **BILLABLE CODE**
- C10.2 - Malignant neoplasm of lateral wall of oropharynx **BILLABLE CODE**
- C10.3 - Malignant neoplasm of posterior wall of oropharynx **BILLABLE CODE**
- C10.4 - Malignant neoplasm of branchial cleft **BILLABLE CODE**
- C10.8 - Malignant neoplasm of overlapping sites of oropharynx **BILLABLE CODE**
- C10.9 - Malignant neoplasm of oropharynx, unspecified **BILLABLE CODE**

Malignant neoplasm of nasopharynx (C11)

- C11.0 - Malignant neoplasm of superior wall of nasopharynx **BILLABLE CODE**
- C11.1 - Malignant neoplasm of posterior wall of nasopharynx **BILLABLE CODE**
- C11.2 - Malignant neoplasm of lateral wall of nasopharynx **BILLABLE CODE**
- C11.3 - Malignant neoplasm of anterior wall of nasopharynx **BILLABLE CODE**
- C11.8 - Malignant neoplasm of overlapping sites of nasopharynx **BILLABLE CODE**
- C11.9 - Malignant neoplasm of nasopharynx, unspecified **BILLABLE CODE**

Malignant neoplasm of pyriform sinus (C12)

- C12 - Malignant neoplasm of pyriform sinus **BILLABLE CODE**

Malignant neoplasm of hypopharynx (C13)

- C13.0 - Malignant neoplasm of post cricoid region **BILLABLE CODE**
- C13.1 - Malig neoplasm of aryepiglottic fold, hypopharyngeal aspect **BILLABLE CODE**
- C13.2 - Malignant neoplasm of posterior wall of hypopharynx **BILLABLE CODE**
- C13.8 - Malignant neoplasm of overlapping sites of hypopharynx **BILLABLE CODE**
- C13.9 - Malignant neoplasm of hypopharynx, unspecified **BILLABLE CODE**

Malig neoplasm of sites in the lip, oral cavity and pharynx (C14)

- C14.0 - Malignant neoplasm of pharynx, unspecified **BILLABLE CODE**
- C14.2 - Malignant neoplasm of Waldeyer's ring **BILLABLE CODE**

C14.8 – Malignant neoplasm of overlap sites of lip, oral cavity and pharynx **BILLABLE C**

(Homelessness)
Z590

(Problem related to living alone)
Z602
