# Supplementary material for: Predictors for Emergency Admission Among Homeless Metastatic Cancer Patients and Association of Social Determinants of Health with Negative Health Outcomes
Source: Cancers (Basel). 2025 Mar 27;17(7):1121. doi: 10.3390/cancers17071121 (PMC11987736; doi:10.3390/cancers17071121)
Supplement: Supplementary file 1 [file cancers-17-01121-s001.zip › Table Supplem S1. PCa PrbLA Factors.pdf]

Supplementary Table S1. Weighted generalized linear models estimating association between PrbLA and the outcomes: anxiety and depression, and LOS, PCa 2017 NIS (weighted n=209,410)

	**aOR (95% CI)**	**Coefficient and 95% CIs (back transformed from log transformation)**
	**Anxiety and Depression**	**LOS**
**PCa PrbLA status**		
Non- PrbLA	Reference	Reference
PrbLa	2.7 (1.64- 4.46)	1.44 (1.11-1.88)
**Age**	0.99 (0.98–0.99)	1.01 (1.00-1.01)
**RACE (%)**		
White	Reference	Reference
Black	0.57 (0.51-0.63)	1.22 (1.14-1.30)
Hispanic	0.62 (0.54-0.72)	1.07 (0.97-1.18)
Asian and Native American and Other	0.64 (0.53-0.79)	1.06 (0.95-1.18)
**Expected primary payer**		
Medicare	Reference	Reference
Medicaid	1.00 (0.86-1.17)	1.24 (1.09-1.39)
Private insurance	0.63 (0.57-0.69)	0.87 (0.81-0.92)
Self-pay and No charge and Other	0.82 (0.68-0.98)	0.76 (0.64-0.89)
**Patient Location: NCHS Urban-Rural Code**		
Central counties of metro areas of >=1 million population	Reference	Reference
Fringe" counties of metro areas of >=1 million population	0.94 (0.86-1.03)	1.04 (0.97-1.11)
Counties in metro areas of 250,000-999,999 population.	0.94 (0.85-1.03)	1.05 (0.98-1.13)
Counties in metro areas of 50,000-249,999 population.	0.89 (0.79-1.01)	1.07 (0.98-1.16)

Micropolitan counties and Not metropolitan or micropolitan counties	0.85 (0.75-0.93)	0.96 (0.88-1.05)	
**Elixhauser comorbidity score**	0.99 (0.99-1.00)	1.04 (1.04-1.05)	
**Median household income**			
0-25th percentile	Reference	Reference	
26th to 50th percentile	0.97 (0.89-1.07)	0.94 (0.88-0.99)	
51st to 75th percentile	0.98 (0.89-1.08)	0.91 (0.86-0.97)	
76th to 100th percentile	0.94 (0.86-1.05)	0.89 (0.83-0.96)	
**Indicator of a transfer out of the hospital**			
Non-transferred out			Reference
Transferred out			2.21 (2.08-2.34)

Abbreviations: NIS, National inpatient sample; NCHS, National Center for Health Statistics; LTA, long-term aspirin users; CI, Confidence Intervals; aOR, adjusted odds ratio; PCa, prostate cancer; LOS, in-hospital length of stay
