# Supplementary material for: Predictors for Emergency Admission Among Homeless Metastatic Cancer Patients and Association of Social Determinants of Health with Negative Health Outcomes
Source: Cancers (Basel). 2025 Mar 27;17(7):1121. doi: 10.3390/cancers17071121 (PMC11987736; doi:10.3390/cancers17071121)
Supplement: Supplementary file 1 [file cancers-17-01121-s001.zip › Table Supplem S2. Breast PrbLA Factors.pdf]

Supplementary Table S2. Weighted generalized linear models estimating association between PrbLA and the outcomes: anxiety and depression, and LOS, Breast Cancers 2017 NIS (weighted n=117,080)

	aOR (95% CI)	Coefficient and 95% CIs (back transformed from log transformation)
	**Anxiety and Depression**	**LOS**
**Breast Cancer PrbLA status**		
Non- PrbLA	Reference	Reference
PrbLA	2.19 (1.12-4.29)	2.14 (1.63-2.82)
**Age**	0.98 (0.98-0.99)	0.99 (0.99-1.00)
**RACE (%)**		
White	Reference	Reference
Black	0.49 (0.45-0.55)	1.17 (1.08-1.27)
Hispanic	0.57 (0.51-0.65)	0.96 (0.85-1.08)
Asian and Native American and Other	0.41 (0.34-0.49)	0.95 (0.84-1.06)
**Expected primary payer**		
Medicare	Reference	Reference
Medicaid	0.90 (0.79-1.02)	1.09 (0.99-1.22)
Private insurance	0.67 (0.60-0.74)	0.94 (0.87-1.02)
Self-pay and No charge and Other	0.61 (0.49-0.74)	0.92 (0.77-1.10)
**Patient Location: NCHS Urban-Rural Code**		
Central counties of metro areas of >=1 million population	Reference	Reference
Fringe" counties of metro areas of >=1 million population	1.07 (0.98-1.17)	1.01 (0.93-1.11)

Counties in metro areas of 250,000-999,999 population.	1.04 (0.94-1.17)	1.02 (0.93-1.12)
Counties in metro areas of 50,000-249,999 population.	1.00 (0.88-1.17)	1.06 (0.94-1.19)
Micropolitan counties and Not metropolitan or micropolitan counties	0.92 (0.82-1.04)	0.99 (0.90-1.09)
**Elixhauser comorbidity score**	0.99 (0.99-1.00)	1.04 (1.04-1.04)
**Median household income**		
0-25th percentile	Reference	Reference
26th to 50th percentile	0.97 (0.89-1.07)	0.93 (0.86-1.00)
51st to 75th percentile	0.92 (0.83-1.02)	0.89 (0.82-0.97)
76th to 100th percentile	0.91 (0.83-1.02)	0.83 (0.76-0.92)
**Indicator of a transfer out of the hospital**		
Non-transferred out		Reference
Transferred out		2.21 (2.03-2.42)

Abbreviations: NIS, National inpatient sample; NCHS, National Center for Health Statistics; LTA, long-term aspirin users; CI, Confidence Intervals; aOR, adjusted odds ratio; PrbLA, Problems related to living alone; LOS, in-hospital length of stay
