# Supplementary material for: Predictors for Emergency Admission Among Homeless Metastatic Cancer Patients and Association of Social Determinants of Health with Negative Health Outcomes
Source: Cancers (Basel). 2025 Mar 27;17(7):1121. doi: 10.3390/cancers17071121 (PMC11987736; doi:10.3390/cancers17071121)
Supplement: Supplementary file 1 [file cancers-17-01121-s001.zip › Table Supplem S3 . Lung PrbLA Factors.pdf]

Supplementary Table S3. Weighted generalized linear models estimating association between PrbLA and the outcome: LOS, Lung patients stratified to Females, 2017 NIS (weighted n=196,930)

	Coefficient and 95% CIs (back transformed from log transformation)
	**LOS**
**Lung PrbLA status**	
Non- Lung PrbLA	Reference
Lung PrbLA	1.29 (1.02-1.63)
**Age**	0.99 (0.99-1.00)
**RACE (%)**	
White	Reference
Black	1.09 (1.02-1.18)
Hispanic	0.90 (0.79-1.02)
Asian and Native American and Other	1.01 (0.91-1.13)
**Expected primary payer**	
Medicare	Reference
Medicaid	0.92 (0.84-1.01)
Private insurance	0.89 (0.83-0.96)
Self-pay and No charge and Other	0.57 (0.47-0.69)
**Patient Location: NCHS Urban-Rural Code**	
Central counties of metro areas of >=1 million population	Reference
Fringe" counties of metro areas of >=1 million population	0.97 (0.91-1.04)
Counties in metro areas of 250,000-999,999 population.	1.02 (0.94-1.09)
Counties in metro areas of 50,000-249,999 population.	0.97 (0.88-1.06)

Micropolitan counties and Not metropolitan or micropolitan counties	0.89 (0.82-0.96)
**Elixhauser comorbidity score**	1.03 (1.02-1.03)
**Median household income**	
0-25th percentile	Reference
26th to 50th percentile	0.91 (0.86-0.98)
51st to 75th percentile	0.94 (0.88-1.01)
76th to 100th percentile	0.92 (0.85-0.99)

Abbreviations: NIS, National inpatient sample; NCHS, National Center for Health Statistics; LTA, long-term aspirin users; CI, Confidence Intervals; aOR, adjusted odds ratio; PCa, prostate cancer; LOS, in-hospital length of stay
