# Supplementary material for: Predictors for Emergency Admission Among Homeless Metastatic Cancer Patients and Association of Social Determinants of Health with Negative Health Outcomes
Source: Cancers (Basel). 2025 Mar 27;17(7):1121. doi: 10.3390/cancers17071121 (PMC11987736; doi:10.3390/cancers17071121)
Supplement: Supplementary file 1 [file cancers-17-01121-s001.zip › Table Suppleme S4. CLOP Homelessness Factors.pdf]

Supplementary Table S4. Weighted generalized linear models estimating association between homelessness and the outcomes: anxiety and depression, and LOS, CLOP 2017 NIS (weighted n=54,265).

	aOR (95% CI)	Coefficient and 95% CIs (back transformed from log transformation)
	**Anxiety and Depression**	**LOS**
**PCa homelessness status**		
Non- Homelessness	Reference	Reference
Homelessness	2.80 (1.68-4.69)	2.58 (1.17- 3.68)
**Age**	0.98 (0.97–0.98)	0.99 (0.99-0.99)
**Female**	1.63 (1.47-1.81)	
**Expected primary payer**		
Medicare	Reference	Reference
Medicaid	0.79 (0.67-0.93)	1.19 (1.01-1.41)
Private insurance	0.76 (0.67-0.87)	1.14 (1.00-1.29)
Self-pay and No charge and Other	0.67 (0.53-0.86)	1.02 (0.79-1.30)
**Patient Location: NCHS Urban-Rural Code**		
Central counties of metro areas of >=1 million population	Reference	Reference
Fringe" counties of metro areas of >=1 million population	1.07 (0.93-1.23)	1.04 (0.89-1.2)
Counties in metro areas of 250,000-999,999 population.	1.18 (1.02-1.36)	1.05 (0.92-1.19)
Counties in metro areas of 50,000-249,999 population.	1.25 (1.01-1.54)	0.99 (0.83-1.17)
Micropolitan counties and Not metropolitan or micropolitan counties	1.14 (0.96-1.35)	0.86 (0.74-1.01)
**Elixhauser comorbidity score**	0.99 (0.98-0.99)	1.04 (1.03-1.04)
**Median household income**		
0-25th percentile	Reference	Reference
26th to 50th percentile	0.97 (0.84-1.1)	0.89 (0.79–1.00)
51st to 75th percentile	0.92 (0.80-1.06)	0.79 (0.69–0.91)
76th to 100th percentile	0.97 (0.83-1.14)	0.74 (0.63–0.86)
**Indicator of a transfer out of the hospital**		
Non-transferred out		Reference

Transferred out	2.2 (1.97-2.51)
